# An interactive class-agnostic detection framework for generalizable colony counting

**DOI:** 10.3389/fmicb.2026.1967912

**Published:** 2026-10-21

**Authors:** Jingxing Fan, Jinrong Shen, Yanqi Zhu, Pangen Hu, Haofeng Li, Gangwei Xu, Zhehao Zhao, Dongping Wu

**Affiliations:** 1 State Key Laboratory of Integrated Chips and Systems, College of Integrated Circuits and Micro-Nano Electronics, Fudan University, Shanghai, China; 2 Shanghai Institute of Infectious Disease and Biosecurity, School of Basic Medical Sciences, Fudan University, Shanghai, China; 3 Shanghai Municipal Center for Disease Control and Prevention, Shanghai, China; 4 Lingnan University, Tuen Mun, New Territories, Hong Kong SAR, China; 5 Shanghai Turtle Technology Co. Ltd., Shanghai, China

**Keywords:** dataset annotation tool, human-computer interaction, interactive object detection, microbial colony counting, microbial colony detection

## Abstract

**Introduction:**

Microbiological monitoring is an essential component of laboratory and industrial quality-control workflows, yet accurate colony counting remains challenging to generalize across diverse colony types and imaging conditions. To address this challenge, we propose the Interactive Colony Counter (ICC), a lightweight and class-agnostic detection framework for microbial colony counting.

**Methods:**

Aiming to improve generalization across diverse conditions, ICC takes the entire colony plate image as input and iteratively integrates user guidance, in the form of bounding boxes for target colonies and corrections of detection errors. ICC consists of three components: the Cross Stage Partial Fusion Network (CSPFNet) as the backbone, the Contrastive-Guidance Encoder (CGE) for processing user guidance, and the Multi-scale Detection Module (MDM) as the detection head. By training on the AGAR dataset with a class-agnostic strategy, ICC learns general colony features rather than relying on type-specific labels.

**Results:**

Under simulated user guidance, experimental results show that ICC achieves competitive performance on the AGAR dataset, with a mean Average Precision (mAP) at IoU 0.5:0.95 of 60.7 and 62.9% at different resolutions. To assess generalization beyond the training domain, ICC is further evaluated on the ADBC dataset under different imaging conditions, which also includes species not represented in the training data. In addition, we demonstrate the feasibility of ICC-assisted annotation by constructing the *B. lactis*-Colony dataset for colony detection, which is generated from *Bifidobacterium lactis* and constructed using the ICC-assisted annotation workflow.

**Discussion:**

Overall, ICC demonstrates strong potential for colony detection and dataset annotation, providing an effective lightweight framework with iterative user guidance for microbiological imaging tasks.

## 1 Introduction

Bacterial colony counting is a critical procedure in microbiological monitoring, widely applied in pharmaceutical production (Majchrowska et al., 2025), environmental monitoring (Zhang et al., 2022) and food safety (Garcia-Armesto et al., 1993). Plate-based colony counting remains widely used in laboratory and industrial settings because of its low cost and ease of implementation. Nevertheless, traditional counting methods remain labor-intensive and time-consuming (Zhang et al., 2022), highlighting a crucial need for a robust and generic automated colony counter.

Conventional image processing methods often struggle to generalize across varying imaging conditions when applied to colony images. To improve the efficiency and consistency of colony analysis, many end-to-end imaging systems covering acquisition, processing, colony detection and counting have been proposed (Zhang et al., 2008; Clarke et al., 2010; Rodrigues et al., 2022; Brugger et al., 2012). Most of these approaches rely on thresholding to segment colony regions (Zhang et al., 2008; Clarke et al., 2010; Sieuwerts et al., 2008) or circle detection to locate the targets (Rodrigues et al., 2022; Brugger et al., 2012), which limits their ability to robustly recognize diverse colonies and makes the results dependent on human expertise. A major factor limiting the adoption of these systems is the high cost of standardized imaging systems, which conflicts with the affordability and simplicity that make colony counting attractive in the first place.

With the development of neural networks and deep learning methods in computer vision, image segmentation and object detection techniques have become increasingly popular. Since colony counting can be formulated as either a segmentation (Minaee et al., 2022) or detection (Zou et al., 2023) task, it is natural to expect the appearance of automated colony counters in this area. A straightforward yet effective approach is to directly predict the number of colonies in an image, often using a tile-based strategy (Ferrari et al., 2015; Hsiao et al., 2025), where each tile is counted individually. Alternatively, the transferred CSRNet (Li et al., 2018; Albaradei et al., 2020) has been employed to generate density maps of colonies across entire plate images. Another common strategy is to first perform segmentation, using methods such as DBSCAN clustering (Zhang, 2022), ResNet-based U-Net (Beznik et al., 2022), c-ResUNet (Morelli et al., 2021), or SegNet (Sannidhan et al., 2024), and then derive colony numbers from the generated masks. Object detection models have also been explored for localizing individual colonies and subsequently computing counts. For instance, PetiteFinder (Nunn et al., 2023) leverages a Faster R-CNN (Ren et al., 2016) architecture built upon a ResNet-50 backbone (He et al., 2016) with a feature pyramid network (FPN) (Lin et al., 2017), specifically targeting the detection of Petite and Grande colonies. In addition, with the introduction of the AGAR dataset (Majchrowska et al., 2025), several models, including YOLOv5 (Whipp and Dong, 2022), Faster R-CNN, and Cascade R-CNN (Cai and Vasconcelos, 2018) with diverse backbones (He et al., 2016; Wang et al., 2020) have been tested on this benchmark. However, most of these deep learning–based methods exhibit limited generalizability and are typically validated only on specific colony types under consistent imaging conditions. In addition, they often require tile-based processing to handle high-resolution colony images.

Beyond automated colony analysis, human–computer interaction has increasingly been used to improve the robustness and generalization ability of neural networks. Interactive methods have become increasingly popular in segmentation tasks. Besides textual prompts (Ding et al., 2020; Kirillov et al., 2023), a widely adopted interactive strategy is the use of human clicks to indicate regions of interest in the image, enabling more flexible and efficient model guidance. Attention mechanisms that emphasize either the first click (Lin et al., 2020) or newly added clicks (Chen et al., 2022) have been proposed to leverage human input effectively. In addition, there are several methods incorporating the previous prediction mask as part of model input (Chen et al., 2022; Sofiiuk et al., 2022; Liu et al., 2023), enabling an iterative pipeline that refines results. Notably, interactive methods have also been successfully applied to medical image segmentation (Zhang et al., 2024), suggesting that interactivity may help models generalize better to diverse conditions. In contrast to interactive segmentation, interactive object detection has received less attention. This is mainly because detection methods predict bounding boxes rather than masks, making it more challenging for models to incorporate human clicks. Nevertheless, several studies have explored the use of human clicks to enhance object detection performance (Lee et al., 2022; Choi et al., 2024), illustrating the potential of interactive object detection strategies.

In summary, deep learning–based colony counting faces three major challenges. First, the availability of sufficiently diverse training datasets remains limited. Compared with conventional object detection tasks, it is difficult to label colony datasets due to the variations in colony number and density. Second, conventional detectors often exhibit limited generalization, especially when applied to unexpected imaging conditions or unseen colony types. Unlike datasets for general object detection tasks in the real world, colony datasets are rare and far from being as comprehensive as large-scale benchmarks like ImageNet (Deng et al., 2009). Traditional models, which are trained on datasets that do not adequately represent the diversity of possible conditions, tend to perform poorly when confronted with unforeseen scenarios. Third, whole-plate colony images are typically acquired at high resolutions, resulting in high-resolution images that often exceed 2000 pixels and can reach up to 4,000 pixels. Directly processing such large images incurs substantial computational costs for conventional methods, while tile-based strategies introduce additional processing steps and increase the risk of error. Moreover, a smaller input resolution inherently limits the effective receptive field, thereby weakening the model’s ability to capture global context and constraining its generalization.

Given the above challenges, it is essential to improve the model’s ability to generalize to previously unseen colony types and diverse imaging conditions, while maintaining a lightweight architecture. Introducing user guidance is particularly well suited to this scenario, as it allows the model to incorporate additional prior knowledge beyond that obtained from purely data-driven training. Specifically, user-provided inputs can guide the model toward ambiguous or error-prone regions, effectively reducing uncertainty caused by previously unseen colony types or varying imaging conditions. Moreover, colony counting inherently involves visually distinct and localized targets, making such interactions easy for users to perform. In parallel, class-agnostic training further enhances model generalization by preventing over-reliance on specific colony types present in the training data.

In this work, we propose the Interactive Colony Counter (ICC; workflow shown in Figure 1), a lightweight object detection framework that integrates human–computer interaction for microbial colony analysis. ICC combines an iterative interaction strategy with class-agnostic training to improve adaptability to diverse imaging conditions and previously unseen colony types. The architecture is further designed to process high-resolution whole-plate images directly. We evaluate ICC on multiple datasets to assess its detection accuracy, counting performance, generalization ability, and transferability. To further demonstrate its practical utility for dataset annotation, we construct the *B. lactis*-Colony dataset using the ICC-assisted annotation workflow.

**Figure 1:**
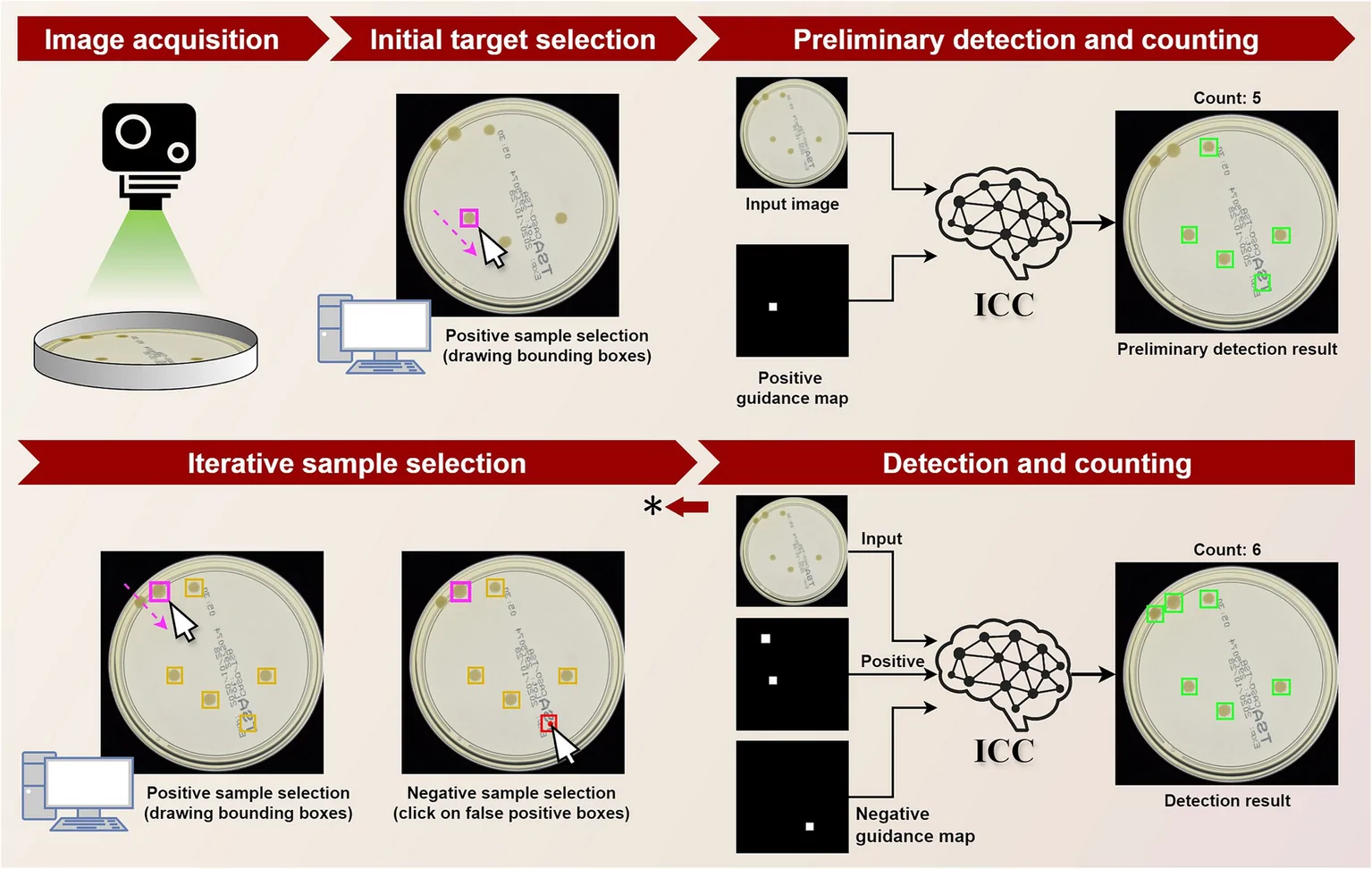
Overview of the Interactive Colony Counter (ICC) workflow. *Indicates iterative user–model interaction.

## 2 Materials and methods

### 2.1 Datasets and preprocessing

We employed the AGAR dataset (Majchrowska et al., 2025) to train and evaluate our Interactive Colony Counter (ICC), as it provides a large number of samples and diverse colony morphologies under varying imaging conditions. The dataset comprises images of five representative microbial species—*S. aureus*, *B. subtilis*, *P. aeruginosa*, *E. coli*, and *C. albicans*—and contains images acquired under four imaging conditions: bright illumination, dark illumination, vague appearance, and reduced resolution. In addition, this dataset is divided into two major subsets: a higher-resolution subset (~4,000 × 6,000 px) and a lower-resolution subset (~2048 × 2048 px). Both subsets are included in the training set, with the higher-resolution images first resized to 2048 × 2048 px. We followed the original AGAR image partition without further re-splitting the dataset. Specifically, 11,934 images were used for training and 3,978 images were used for validation. Since the original image partition does not provide an independent test set, the AGAR results reported in this study should be interpreted as validation performance. Among the 60 training epochs, the checkpoint with the highest validation mAP was selected for evaluation. In addition, we applied data augmentations during training in order to improve robustness, including conversion to grayscale, random flips, cropping, and adjustments of brightness and contrast.

For evaluating the generalization of ICC, we employed the ADBC dataset (Makrai et al., 2023), which is well-suited for evaluation and not included in our training set. This dataset contains images of 24 different bacterial species cultured on various agar media. Most images were captured using consumer-grade mobile phones under typical laboratory conditions (~2,700 × 2,700 px), resulting in noticeable reflective artifacts. This dataset was therefore used as an external benchmark under different imaging conditions and additionally includes species not represented in the training data. Notably, ADBC was used only for external evaluation and did not influence model training, checkpoint selection, confidence-threshold selection, or other analysis settings.

In addition, we incorporated the TXL-PBC dataset (Gan et al., 2025), a curated and re-annotated peripheral blood cell dataset (~256 × 256 px) compiled from four publicly available sources. This dataset allows us to evaluate the transferability of ICC to another cell-level detection task.

To evaluate the practical utility of ICC as an annotation tool, we constructed the *B. lactis*-Colony dataset (examples in Figure 2A), which was generated from *Bifidobacterium lactis* (detail workflow described in Section S2) and annotated using the ICC-assisted workflow. This dataset consists of 288 plate images captured under diverse conditions, including bright and dark illumination as well as higher-resolution (3,024 × 3,024 px) and lower-resolution (2,700 × 2,700 px) settings. It contains two types of colonies: *B. lactis* colonies and non-target lactic acid bacteria–like colonies (non-target LAB, count distribution in Figure 2C), with the two categories assigned based on colony appearance and neither type appears in the training set of ICC.

**Figure 2:**
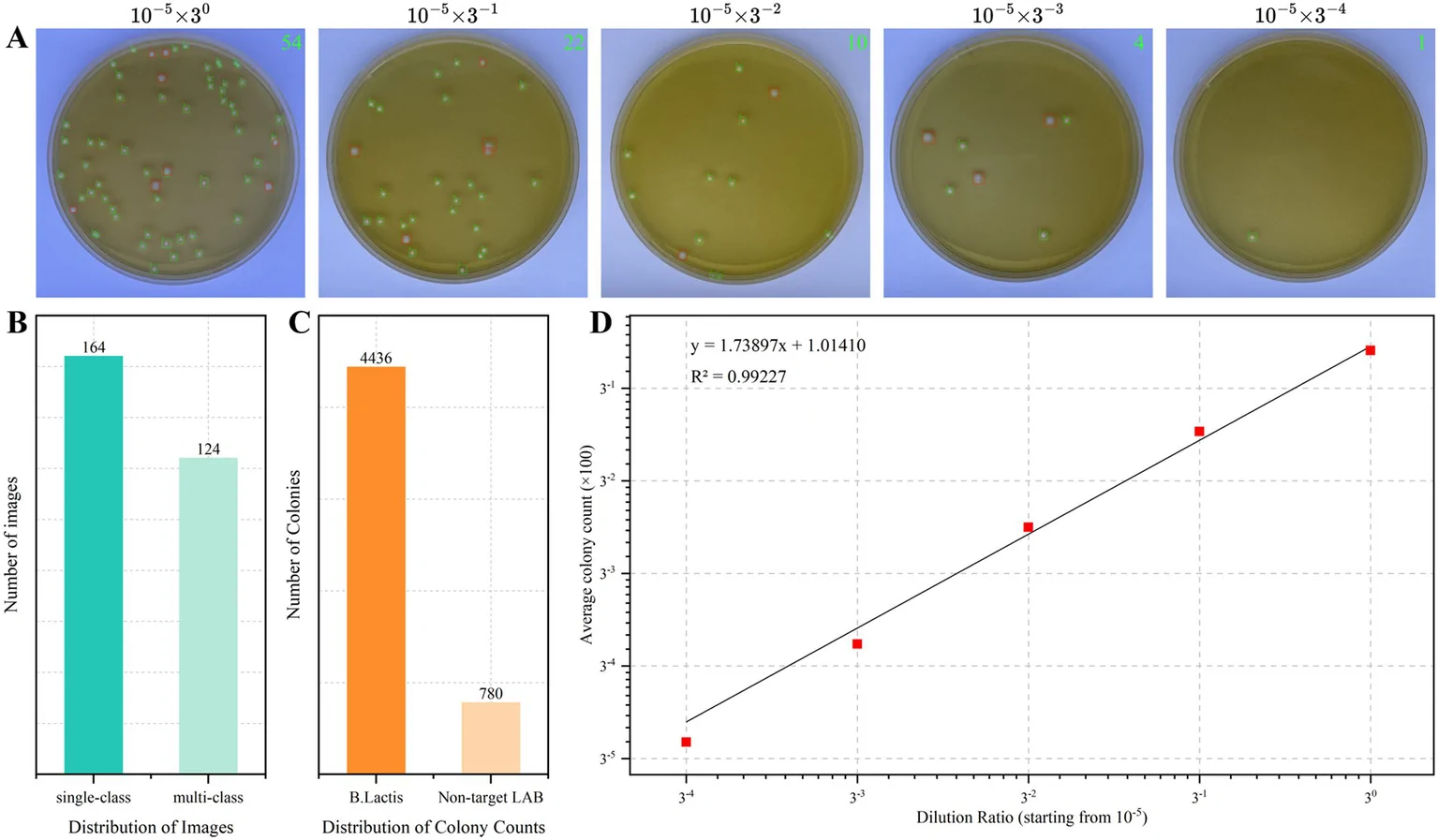
Concentration-gradient control experiment for the *B. lactis*-Colony dataset. **(A)** Example plate images at each concentration, ranging from 10^−5^ × 3^0^ to 10^−5^ × 3^−4^. **(B)** Distribution of images by number of classes in the entire dataset. **(C)** Distribution of colony counts by categories in the entire dataset. **(D)** The fitted result of concentration-gradient control experiment, where each data point represents the average of ten replicates.

Our method was implemented in Python 3.8.1 using PyTorch 2.0.1 and NVCC 11.8. The neural network was trained and evaluated on a desktop with an Intel Xeon Gold 6,133 CPU and an NVIDIA GeForce RTX 4090 GPU.

### 2.2 Overview of ICC architecture

The overall architecture of ICC is shown in Figure 3. ICC comprises three components: Cross Stage Partial Fusion Network (CSPFNet, Figure 3A), Contrastive-Guidance Encoder (CGE, Figure 3B) and Multi-scale Detection Module (MDM, Figure 3C). Given a colony image and the corresponding user guidance, the grayscale colony image is fed into CSPFNet, while the user input is encoded by CGE. CGE acts as a down-sampling encoder for representation learning, where the features of positive and negative inputs are concatenated and then integrated into the corresponding layers of CSPFNet. Within the backbone, image features and human guidance are fused to obtain the target colony representations. Subsequently, high-level features are sent to MDM, which performs multi-scale detection for the target colony type. In addition, skip connections from early stages of CSPFNet are incorporated to mitigate the loss of fine-grained image features.

**Figure 3:**
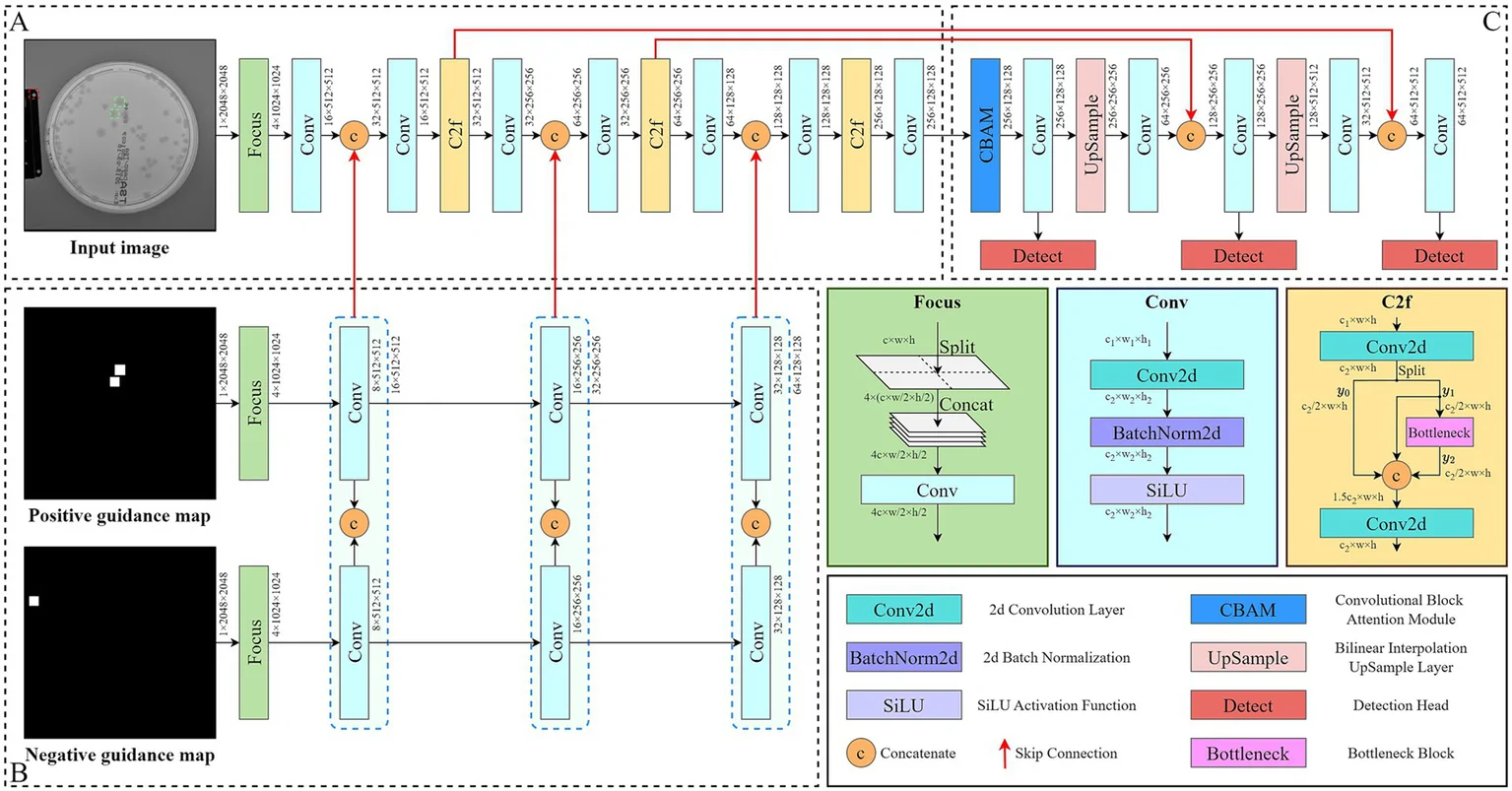
Overview of ICC architecture. **(A)** CSPFNet: backbone for feature extraction and fusion. **(B)** CGE: encoder for human-guided representation learning. **(C)** MDM: multi-scale detector for class-agnostic colony detection.

### 2.3 Cross stage partial feature fusion

In our backbone CSPFNet, the network is designed not only to extract features from colony images, but also to fuse guidance features provided by user inputs. To preserve fine-grained image features while handling high-resolution colony images, the backbone begins with a Focus layer (Focus in Figure 3), which takes a 2048 × 2048 × 1 px input. This layer slices the input along the spatial dimension and re-arranges it into the channel dimension, thereby reducing the spatial resolution while maintaining feature details. In addition, channel-wise reorganization is more efficient than spatial convolutions, as it shifts computation from spatial to the channel dimension.

To reduce spatial dimensions and perform channel-wise computation, a convolutional layer (Conv in Figure 3) is applied following the Focus layer and also after concatenation. Here, we adopt the SiLU activation function instead of conventional ReLU. SiLU has been shown to provide smoother gradients and improved performance in deep models, particularly for detection tasks (Li et al., 2022; Wang et al., 2023; Terven et al., 2023). The SiLU activation can be formulated as Equation 1:

$$\text{SiLU}(x)=x\cdot \sigma (x)=\frac{x}{1+e^{-x}}$$ (1)

Compared to ReLU, SiLU avoids the zero-gradient issue by allowing small negative activations. As a result, feature expressiveness and stabilizing optimization have been enhanced.

Notably, user guidance features are fused with image features via concatenation rather than addition, preserving the original image-feature channels while introducing complementary guidance information. We employ the Cross Stage Partial Bottleneck with Two Convolutions (C2f, Figure 3, C2f) (Terven et al., 2023) for the further refinement of these fused features. This module focuses on channel-wise computation, enabling efficient feature reuse and thereby enhancing the sensitivity of the network to subtle variations in colony patterns.

### 2.4 Contrastive-guidance input

In interactive segmentation, human input is typically encoded as click maps, which serve as spatial cues on the feature map (Lin et al., 2020; Chen et al., 2022; Sofiiuk et al., 2022; Liu et al., 2023). Such inputs are well suited for segmentation tasks, as clicks can be labeled as positive (foreground) or negative (background), providing guidance for the segmentation process. Moreover, click-based interaction involves relatively low human effort.

In this work, we employ user-provided bounding boxes as an alternative form of human-computer interaction (Figures 4B,C). While serving a similar role as point-based clicks in indicating the target of interest, bounding boxes additionally capture surrounding context, which can be incorporated into the guidance features. Drawing a bounding box requires a drag operation rather than a single click, but provides additional spatial and contextual information and is naturally aligned with the output representation of object detectors. This alignment simplifies the model’s interpretation of bounding boxes and supports iterative interactions, such as selecting incorrectly predicted boxes for correction. Iteration in our interaction strategy is achieved through iterative user actions: in each round, users can select false positive boxes for correction, and add bounding boxes for missed targets.

**Figure 4:**
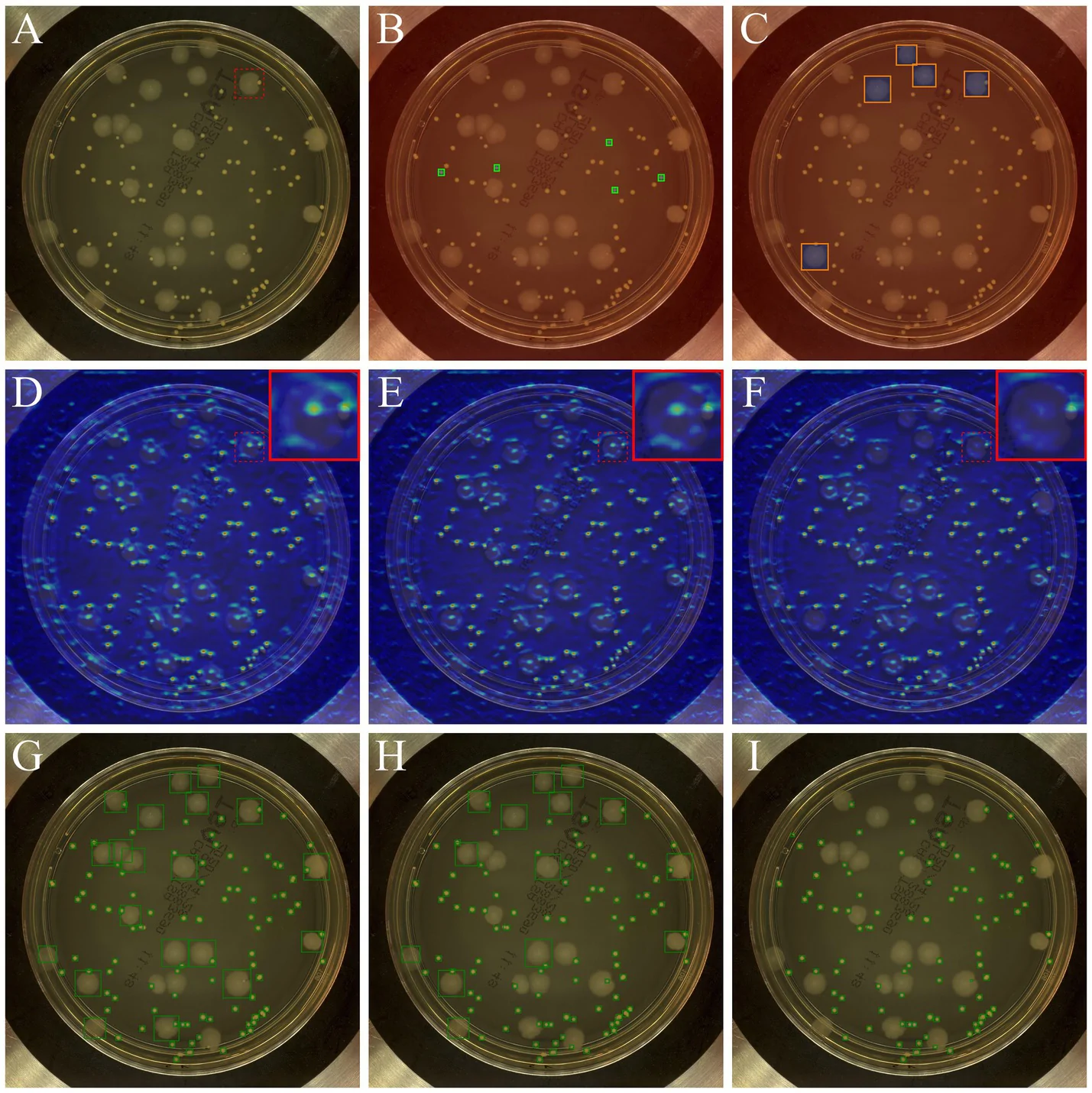
Feature map visualization and detection results. **(A)** Input image. **(B)** Positive guidance map. **(C)** Negative guidance map. **(D–F)** Grad-CAM heatmaps with no user input, positive input, and both inputs, respectively. **(G–I)** Detection results corresponding to **(D–F)**. The source image was obtained from the AGAR dataset (Majchrowska et al., 2025), licensed under CC BY-NC 2.0.

To demonstrate the necessity of iterative interaction and contrastive inputs (positive and negative), we visualized the regions model focused on during inference using Grad-CAM (Selvaraju et al., 2017). Specifically, let $A^{k}$ denote the k -th channel in the feature map before the detection head and y the predicted logit of the target. We first compute the gradient of y with respect to $A^{k}$ , and then apply global average pooling over the spatial dimensions to obtain the channel-wise weight:

$$\alpha_{k}=\frac{1}{H\times W}\sum_{i=1}^{H}\sum_{j=1}^{W}\frac{\partial y}{\partial A_{\mathrm{ij}}^{k}}$$ (2)

In Equation 2, H , W denote the spatial height and width of the feature map. Let C denotes the number of channels, the Grad-CAM heatmap is then computed as:

$$L_{\text{Grad}-\mathrm{CAM}}=\text{ReLU}(\sum_{k=1}^{C}\alpha_{k}A^{k})$$ (3)

Equation 3 highlights the regions of the feature map that contribute most strongly to the prediction, where brighter areas indicate higher relevance. Figure 4 shows an example for detection of smaller colonies (*S. aureus*) in the presence of larger ones (*P. aeruginosa*). With user guidance—either via a positive guidance map (Figure 4B) or both positive and negative guidance maps (Figure 4C)—the model reduces its focus on irrelevant regions while maintaining attention on relevant targets (Figures 4D–F).

### 2.5 Multi-scale detector

In order to enhance the feature representation of CSPFNet, we employ the Convolutional Block Attention Module (CBAM) (Woo et al., 2018) at the early stage of the Multi-scale Detection Module (MDM). CBAM applies attention in both the channel and spatial dimensions, enabling the network to emphasize relevant information while suppressing background or irrelevant features. Consequently, the model can better focus on regions that contribute to object detection, which is important in complex scenes.

Inspired by YOLOv5, we incorporate upsampling and skip connections after the CBAM module, enabling a multi-scale prediction strategy. Therefore, features from different resolutions are fused, and predictions are generated at multiple scales. The shallow layers mainly capture detailed information for fine-grained, low-level features, while the deeper layers capture semantic context for high-level features. As a result, the multi-scale detection module improves robustness in scenarios with significant variation in image conditions or colony types.

### 2.6 Simulated user interaction

To reduce dependence on colony types represented in the training set, we adopted a class-agnostic training strategy by removing class labels during training, treating all colony types as a single category. However, under this setup, it is necessary for user guidance to specify which type of colonies should be detected while processing images containing mixed colonies on a single plate. Without such guidance, the model may detect all visible colonies as a single category, leading to ambiguous results.

In practice, user inputs are often shown to be unpredictable and vary across different cases. Therefore, it is necessary to simulate user inputs randomly during training, ensuring that the model can handle user-provided guidance in different situations. Moreover, our method allows users to provide corrective inputs for each prediction iteration, enabling the model to adjust its subsequent predictions accordingly.

Let $G=\{g_{1},g_{2},g_{3},\ldots ,g_{N}\}$ denote the ground truth of an image, consisting of N bounding boxes, and let $U_{t}=(U_{t}^{+},U_{t}^{-})$ represent the user input at the t -th stage of iterative training, which consists of a positive guidance map $U_{t}^{+}$ and a negative guidance map $U_{t}^{-}$ . The prediction of the model at the t -th stage is denoted as $P_{t}=\{p_{1},p_{2},p_{3},\ldots ,p_{M}\}$ , consisting of M bounding boxes.

In the initial simulation stage ( t=0 ), only positive guidance map is provided, since no prediction results are available. The initial simulation of user input $U_{0}$ for the model is defined as Equation 4:

$$\left\{ \begin{array}{c} U_{0}^{+}\subseteq G,|U_{0}^{+}|=\alpha |G|,\alpha \in (0,\frac{1}{2}]\\ U_{0}^{-}=\emptyset \end{array}\right.$$ (4)

Consequently, we can get the prediction result at stage t=0 . In order to simulate the negative guidance maps, the incorrect parts of prediction result at t -th stage are defined as Equations 5, 6:

$$FP_{t}=\{p\in P_{t}|\mathrm{IoU}(p,g)<\tau ,\forall g\in G\}$$ (5)

$$FN_{t}=\{g\in G|\mathrm{IoU}(p,g)<\tau ,\forall p\in P_{t}\}$$ (6)

Where IoU represents Intersection over Union (IoU), and τ means the IoU threshold. In addition, False Positives (FP) denote background regions incorrectly identified as colonies, and False Negatives (FN) denote colonies that were missed. At stage t=1 , only negative guidance map is simulated, while the positive input remains unchanged. The current input can be defined as Equation 7:

$$\left\{ \begin{array}{c} U_{1}^{+}=U_{0}^{+}\\ U_{1}^{-}\subseteq FP_{0},|U_{1}^{-}|=\beta |FP_{0}|,\beta \in (0,\frac{1}{2}] \end{array}\right.$$ (7)

For stage t=2,3,4 , user input is updated by randomly sampling either from $FP_{t-1}$ or $FN_{t-1}$ . For example, a sample s can be randomly selected from $FN_{t-1}$ and added to $U_{t}^{+}$ as Equation 8:

$$\left\{ \begin{array}{c} U_{t}^{+}=U_{t-1}^{+}\cup \{s\},s\sim \text{Uniform}(FN_{t-1}),FN_{t-1}\neq \emptyset \\ U_{t}^{-}=U_{t-1}^{-} \end{array}\right.$$ (8)

Or a sample s can be randomly selected from $FP_{t-1}$ and added to $U_{t}^{-}$ as Equation 9:

$$\left\{ \begin{array}{c} U_{t}^{+}=U_{t-1}^{+}\\ U_{t}^{-}=U_{t-1}^{-}\cup \{s\},s\sim \text{Uniform}(FP_{t-1}),FP_{t-1}\neq \emptyset \end{array}\right.$$ (9)

At each stage, either the positive or negative input is updated, ensuring that the user input changes once per iteration to simulate realistic user interaction. Since the model is also expected to perform without any user input, an external stage is introduced at the beginning of training on a new image, during which no simulated user input is provided.

### 2.7 Loss function

We adopt box regression loss and objectness (confidence) loss as the training objectives. Since all targets belong to a single category, the classification loss is omitted in our method. The overall loss is defined as Equation 10:

$$L=\lambda_{\mathrm{box}}\cdot L_{\mathrm{box}}+\lambda_{\mathrm{obj}}\cdot L_{\mathrm{obj}}$$ (10)

Where $\lambda_{\mathrm{box}}$ and $\lambda_{\mathrm{obj}}$ denote the weights of the box regression loss and objectness loss, respectively. These two terms are formulated as Equations 11, 12:

$$L_{\mathrm{box}}=\frac{1}{N}\sum_{i=1}^{N}{\left(1-\text{CIoU}(b_{i},{\hat{b}}_{i})\right)}^{2}$$ (11)

$$L_{\mathrm{obj}}=\frac{1}{M}\sum_{j=1}^{M}\mathrm{BCE}(s_{j},{\hat{s}}_{j})$$ (12)

Where N is the number of matched predicted boxes, $b_{i}$ and ${\hat{b}}_{i}$ denote the i -th ground truth and predicted bounding boxes; M is the number of objectness predictions, $s_{j}$ is the ground truth objectness and ${\hat{s}}_{j}$ is the corresponding predicted objectness. For a matched object, $s_{j}$ is defined as Equation 13:

$$s_{j}=\beta +(1-\beta )\cdot \mathrm{IoU}(b_{j},{\hat{b}}_{j})$$ (13)

Where β denotes the baseline factor, ensuring that higher-IoU predictions are assigned higher objectness targets, while lower-IoU ones still receive small positive values, making the learning of objectness smoother. For unmatched objects, $s_{j}$ is set to 0. Meanwhile, Complete IoU (CIoU) (Zheng et al., 2020) and Binary Cross Entropy (BCE) are adopted in our method to compute these losses.

## 3 Results

### 3.1 Evaluation metrics

To evaluate the performance of ICC for colony detection, we adopt metrics commonly used in object detection: precision, recall, mean Average Precision (mAP) and F1 score (Everingham et al., 2010). Let True Positives (TP) denote correctly detected colonies. False Positives (FP) and False Negatives (FN) are defined as in Section 2.6. The metrics are then computed as Equations 14, 15, 16:

$$P=\frac{\mathrm{TP}}{\mathrm{TP}+\mathrm{FP}}$$ (14)

$$R=\frac{\mathrm{TP}}{\mathrm{TP}+\mathrm{FN}}$$ (15)

$$F1=\frac{2\mathrm{PR}}{P+R}$$ (16)

Here, P and R represent precision and recall, respectively. Precision, defined as the ratio of correctly identified colonies to all detections, and recall, defined as the ratio of colonies correctly detected among all ground-truth colonies, are balanced using the F1 score—the harmonic mean of these two measures. Additionally, the mean Average Precision (mAP) is calculated from the area under the Precision–Recall (PR) curve, providing a comprehensive assessment of detection performance across varying confidence thresholds.

We have also adopted Mean Absolute Error (MAE) and symmetric Mean Absolute Percentage Error (sMAPE) to evaluate the performance of ICC to counting colonies. Let N denote the number of evaluation images, $c_{i}$ denote the true count of the i -th image and ${\hat{c}}_{i}$ denote the corresponding predicted count. The metrics are computed as Equations 17, 18:

$$\mathrm{MAE}=\frac{1}{N}\sum_{i=1}^{N}|c_{i}-{\hat{c}}_{i}|$$ (17)

$$\text{sMAPE}=\frac{100\%}{N}\sum_{i=1}^{N}\frac{|c_{i}-{\hat{c}}_{i}|}{(|c_{i}|+|{\hat{c}}_{i}|)/2}$$ (18)

### 3.2 Detection and counting performance on the AGAR dataset

We conducted a series of experiments to evaluate the performance of ICC. The model was evaluated under multiple simulated user-interaction iterations, in which guidance was generated as described in Section 2.6. Faster R-CNN (Ren et al., 2016) and Cascade R-CNN (Cai and Vasconcelos, 2018) were included as baseline models, both of which have been shown to be effective in colony counting (Majchrowska et al., 2025).

ICC was trained using the training samples from both the higher- and lower-resolution subsets of the AGAR dataset (Majchrowska et al., 2025), thereby enabling the model to be effective for different resolutions. The training parameters are summarized in Supplementary Table S6. To validate the effectiveness of bounding-box user guidance, we additionally trained a variant of ICC that uses key points derived from simulated user clicks. For clarity, the bounding-box version is denoted as ICC-Bbox, and the key-point version as ICC-Kpt. Both versions were evaluated on the subsets separately (Tables 1, 2). Notably, ICC-Bbox improved on both AGAR subsets after two rounds of user guidance (Tables 1, 2). The published Faster R-CNN and Cascade R-CNN results are shown as AGAR benchmark references. The detailed results are presented in Supplementary Tables S1, S2. Since the Faster R-CNN- and Cascade R-CNN-based methods are not publicly available, we refer to the results reported in Majchrowska et al. (2025).

**Table 1:** Detection performance comparison on the higher-resolution subset of the AGAR dataset.

Methods	Average mAP @[0.50:0.95]							
	Faster R-CNN	Cascade R-CNN	ICC-Kpt			ICC-Bbox		
			Iter 0	Iter 2	Iter 5	Iter 0	Iter 2	Iter 5
*S. aureus*	59.8%	65.6%	49.4%	49.5%	49.2%	56.7%	60.2%	62.6%
*B. subtilis*	40.6%	42.1%	56.7%	58.1%	60.3%	62.3%	64.4%	66.0%
*P. aeruginosa*	44.4%	46.9%	48.4%	51.9%	54.5%	54.6%	59.0%	61.3%
*E. coli*	40.7%	43.1%	54.5%	57.8%	59.3%	59.9%	63.4%	64.6%
*C. albicans*	61.1%	62.3%	32.6%	33.6%	34.1%	40.9%	46.5%	49.2%
Overall	49.3%	52.0%	48.3%	50.2%	51.5%	54.9%	58.7%	60.7%

**Table 2:** Detection performance comparison on the lower-resolution subset of the AGAR dataset.

Methods	Average mAP @[0.50:0.95]							
	Faster R-CNN	Cascade R-CNN	ICC-Kpt			ICC-Bbox		
			Iter 0	Iter 2	Iter 5	Iter 0	Iter 2	Iter 5
*S. aureus*	66.5%	69.2%	54.9%	56.3%	56.1%	53.7%	58.6%	61.6%
*B. subtilis*	41.1%	48.0%	46.6%	52.7%	54.8%	53.0%	57.4%	59.0%
*P. aeruginosa*	50.6%	54.7%	34.4%	51.2%	55.9%	37.1%	58.3%	63.2%
*E. coli*	56.5%	58.2%	45.7%	64.5%	67.6%	47.9%	67.6%	70.5%
*C. albicans*	65.2%	66.8%	52.7%	52.9%	53.0%	54.4%	58.4%	60.1%
Overall	56.0%	59.4%	46.9%	55.5%	57.5%	49.2%	60.1%	62.9%

Given that colony counting focuses on accurate enumeration within individual plates, we evaluated MAE and sMAPE on the detection results of each plate image across the higher- and lower-resolution validation subsets (Tables 3, 4). For the primary counting results, ICC-Bbox was evaluated at predefined interaction budgets of Iter 0, 2, 5, and 10 using a fixed confidence threshold for each resolution subset. The confidence thresholds were selected on the AGAR validation data according to the lowest aggregate sMAPE at Iter 10, resulting in 0.23 for the higher-resolution subset and 0.14 for the lower-resolution subset. MAE and sMAPE were reported from the same evaluation setting. Complete threshold- and iteration-wise results are provided in Supplementary Tables S3, S4.

**Table 3:** Counting performance comparison on the higher-resolution subset of the AGAR dataset.

Methods	MAE & sMAPE*											
	Faster R-CNN		Cascade R-CNN		ICC-Bbox							
					Iter 0		Iter 2		Iter 5		Iter 10	
*S. aureus*	0.42	2.78%	0.68	2.27%	1.50	5.52%	2.25	4.89%	3.00	5.73%	3.50	5.88%
*B. subtilis*	0.66	1.96%	0.68	1.97%	0.88	3.16%	0.25	0.63%	0.25	0.63%	0.25	0.63%
*P. aeruginosa*	1.21	5.53%	1.26	4.48%	13.00	17.13%	12.63	18.85%	8.75	14.48%	1.63	1.37%
*E. coli*	1.62	5.40%	1.58	3.03%	6.38	8.06%	3.38	4.87%	2.88	3.93%	3.00	3.80%
*C. albicans*	0.93	2.98%	0.49	2.05%	5.13	41.28%	2.25	14.09%	1.00	7.29%	1.75	8.39%
Overall	0.97	3.73%	0.94	2.76%	5.38	15.03%	4.15	8.67%	3.18	6.41%	2.03	4.01%

*The left and right columns correspond to MAE and sMAPE, respectively.

**Table 4:** Counting performance comparison on the lower-resolution subset of the AGAR dataset.

Methods	MAE & sMAPE*											
	Faster R-CNN		Cascade R-CNN		ICC-Bbox							
					Iter 0		Iter 2		Iter 5		Iter 10	
*S. aureus*	0.54	3.55%	0.32	2.20%	0.63	1.35%	1.00	1.91%	0.88	1.72%	0.88	1.72%
*B. subtilis*	0.23	0.63%	0.31	0.49%	10.00	13.13%	3.63	4.59%	2.75	3.62%	1.50	2.17%
*P. aeruginosa*	0.55	5.60%	0.40	4.13%	2.25	7.82%	0.88	2.77%	0.75	2.37%	0.38	1.25%
*E. coli*	0.75	4.71%	0.52	3.05%	0.00	0.00%	0.00	0.00%	0.00	0.00%	0.00	0.00%
*C. albicans*	0.37	1.49%	0.21	1.32%	1.63	13.59%	0.38	1.90%	0.38	1.73%	0.38	1.73%
Overall	0.49	3.20%	0.35	2.24%	2.90	7.18%	1.18	2.23%	0.95	1.89%	0.63	1.37%

*The left and right columns correspond to MAE and sMAPE, respectively.

### 3.3 Generalization evaluation on the ADBC dataset

We next evaluated ICC on the ADBC dataset (Makrai et al., 2023), as an external dataset to assess its generalization beyond the training images (Table 5). No ADBC images were included in the training set. Although some species in ADBC are also represented in AGAR, ADBC was acquired under substantially different imaging conditions and additionally contains species not represented in the training data. The dataset also presents more challenging conditions due to frequent reflections and shadows, commonly encountered in laboratory environments. ICC-Bbox with user input achieved an mAP of 59.4% on this dataset, which is considered reasonable given the presence of many challenging imaging conditions and colony appearances.

**Table 5:** Detection performance on the ADBC dataset.

Methods	Average mAP @0.50							
	ICC-Kpt				ICC-Bbox			
Iterations	Iter 0	Iter 2	Iter 5	Iter 10	Iter 0	Iter 2	Iter 5	Iter 10
Overall	45.7%	48.9%	51.4%	54.0%	45.9%	51.0%	54.8%	59.4%

Furthermore, we selected 13 classes with relatively well-defined and individually distinguishable colony morphologies for counting evaluation (as indicated in Supplementary Table S5). Classes showing highly irregular or confluent growth patterns, for which individual-colony counting was less reliable, were not included in this analysis. Accordingly, the aggregate counting results reported in Table 6 apply only to these 13 selected classes.

**Table 6:** Counting performance on the ADBC dataset.

Methods	ICC-Kpt		ICC-Bbox	
	Iter 0	Iter 5	Iter 0	Iter 5
MAE	6.88	6.49	5.01	4.47
sMAPE	17.40%	18.63%	8.43%	8.15%

### 3.4 Transferability to cell-level detection

Finally, we retrained ICC-Bbox on the TXL-PBC dataset (Gan et al., 2025) and tested it with an input size of 640 × 640 × 1 px to evaluate its applicability in cell related medical imaging tasks (Table 7). ICC shows a limited ability to distinguish RBCs and platelets from the background at early iterations, likely because WBCs are far more numerous in this dataset and the model tends to prioritize detecting the most frequent targets. Nevertheless, the performance on these minority classes improves significantly after user interactions. These results suggest that, after retraining on TXL-PBC, ICC-Bbox benefits from iterative user guidance in a microscopic cell-detection task, supporting the potential transferability of the proposed framework to other detection tasks.

**Table 7:** Detection performance on the TXL-PBC dataset.

Methods	ICC-Bbox: mAP & F1 @0.50*					
	Iter 0		Iter 2		Iter 5	
WBC	88.82%	86.30%	94.47%	94.09%	95.62%	95.53%
RBC	7.38%	21.20%	71.44%	57.43%	88.61%	83.04%
Platelets	0.08%	1.56%	62.72%	25.52%	92.01%	65.65%
Overall	32.09%	36.35%	76.21%	59.01%	92.08%	81.41%

*The left and right columns correspond to mAP and F1 score, respectively.

### 3.5 Ablation study and model efficiency

Since ICC incorporates both positive and negative user inputs, it is necessary to evaluate the contribution of each input type. We conducted an ablation study on both AGAR subsets to assess their respective importance (Table 8). In addition, we compare the parameter count and computational complexity of ICC with representative object-detection architectures to contextualize its model size and computational requirements (Table 9).

**Table 8:** Ablation study.

Resolution	Variant	Components enabled	Average mAP & F1 @0.50			
			No user input		With user input	
			mAP	F1	mAP	F1
Higher-resolution	Backbone	CSPFNet+ MDM	89.39%	71.15%	-	-
	With Negative input	CSPFNet+ MDM + Negative Input	69.04%	71.32%	69.92%	73.14%
	With Positive input	CSPFNet+ MDM + Positive Input	87.33%	81.69%	93.31%	82.59%
	Full ICC	CSPFNet+ MDM + CGE	88.12%	83.62%	93.81%	85.97%
Lower-resolution	Backbone	CSPFNet+ MDM	82.05%	72.22%	-	-
	With Negative input	CSPFNet+ MDM + Negative Input	78.50%	80.63%	87.24%	85.64%
	With Positive input	CSPFNet+ MDM + Positive Input	78.60%	82.34%	92.71%	85.59%
	Full ICC	CSPFNet+ MDM + CGE	77.11%	80.98%	95.49%	92.75%

**Table 9:** Model size and computational complexity of ICC and representative methods.

Methods	Input size	Params	FLOPs	FPS
Faster R-CNN	640 × 640	59.1 M	170.2 G	-
YOLOv5n (Jocher, 2020)	640 × 640	1.9 M	4.5 G	-
YOLOv8n (Jocher et al., 2023)	640 × 640	3.2 M	8.7 G	-
YOLO26n (Jocher et al., 2026)	640 × 640	2.4 M	5.5 G	-
ICC	2048 × 2048	2.4 M	20.1 G	64.0
	640 × 640	2.4 M	2.0 G	219.7

## 4 Discussion

### 4.1 Effects of iterative user guidance

The results across the AGAR and ADBC datasets indicate that iterative user guidance generally improves detection performance, although the magnitude of improvement varies across interaction types and evaluation metrics. To assess the effect of iterative user inputs, we plotted PR curves and detection-metric curves from 0 to 10 interaction iterations (Figure 5). The quantitative interaction results were obtained using simulated guidance and a single simulated interaction sequence for each setting. PR curves were generated for both the higher-resolution and lower-resolution subsets of the AGAR dataset using IoU thresholds of 0.5–0.95 and 0.5. Across successive iterations, the PR curves generally shift upward, while the detection metrics show an overall improving trend, indicating the beneficial effect of iterative user guidance under the evaluated simulated sequence.

**Figure 5:**
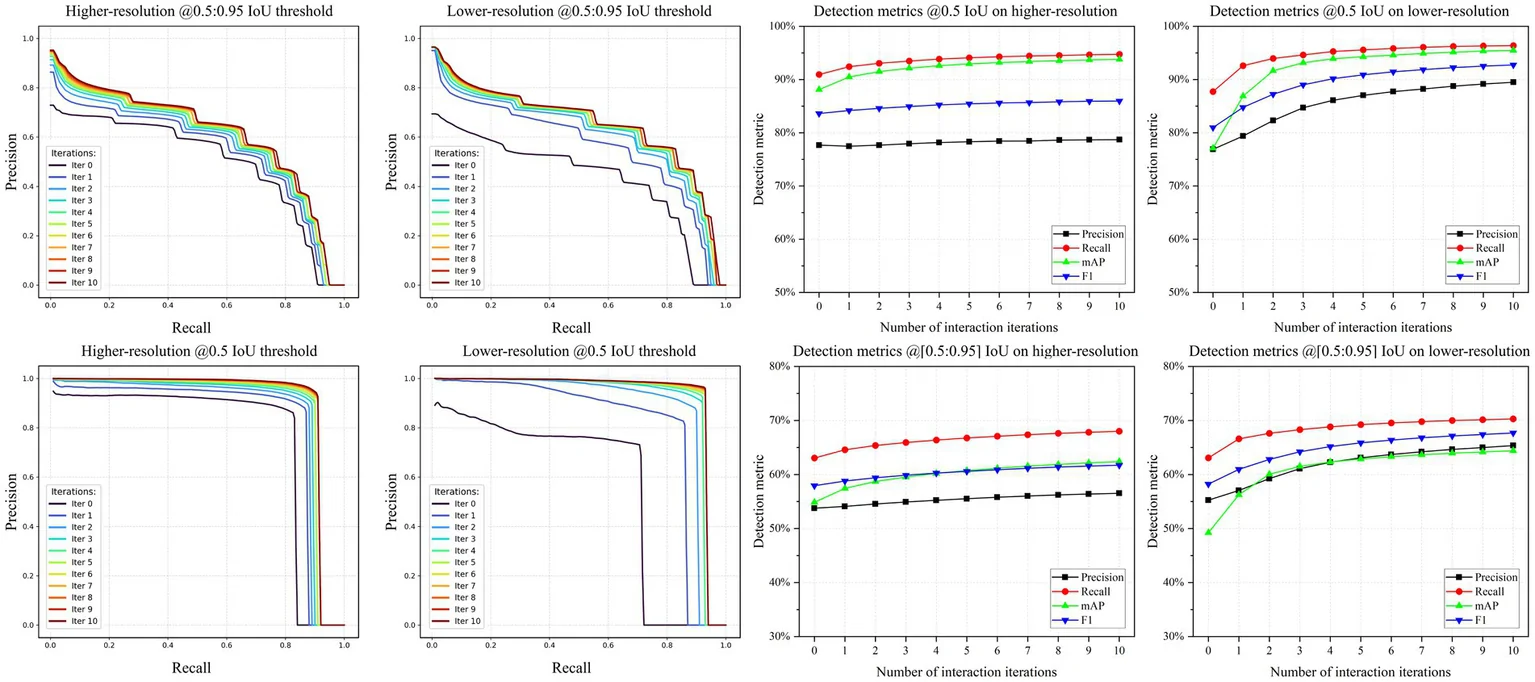
PR curves and detection-metric curves for the higher-resolution and lower-resolution subsets at different IoU thresholds across interaction iterations from 0 to 10.

### 4.2 ICC-assisted colony annotation

We developed an annotation tool for colony datasets using Python Tkinter and integrated ICC into the interface (workflow in Supplementary Figures S1, S2, detailed interface in Supplementary Figure S3). To demonstrate the effectiveness of this tool, we constructed the *B. lactis*-Colony dataset and annotated using the ICC-assisted workflow. To provide an auxiliary consistency check for the constructed dataset, we conducted a concentration-gradient control experiment, where the fitted relationship showed good agreement with the expected concentration-dependent trend (Figure 2D). These results demonstrate the feasibility of ICC-assisted colony annotation and dataset construction.

### 4.3 Limitations in challenging cases

During validation on the AGAR dataset, we observed several challenging cases in which ICC struggled to detect colonies accurately (Figure 6A). Figure 6 presents examples of these cases, where we cropped the relevant regions for clearer illustration. We identified three main scenarios in which ICC is prone to errors: overlapping colonies, colonies with vague boundaries, and colonies with abnormal appearances. In the case of overlapping colonies, user inputs can often correct the detection results because many overlapping colonies still preserve relatively clear boundaries. However, for colonies with vague borders or abnormal appearances, user interactions are much less effective. Colonies with vague borders are sometimes difficult even for humans to delineate, leading to unreliable user guidance. Colonies with abnormal appearances often arise when multiple colonies cluster extremely closely, or when contamination or internal irregularities distort the structure of a single colony.

**Figure 6:**
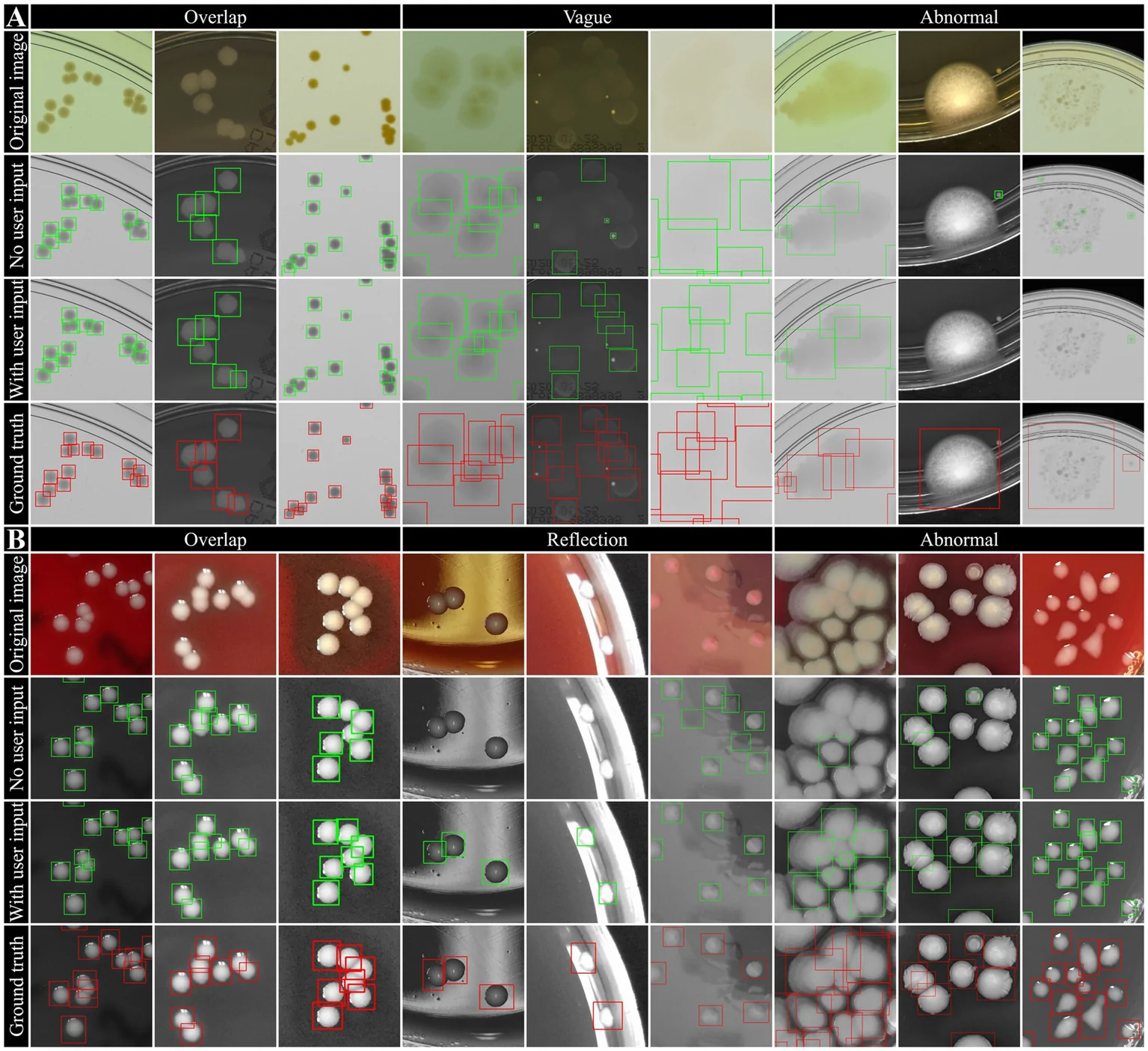
The challenging scenarios for ICC in the AGAR dataset **(A)** and ADBC dataset **(B)**. Source images in **(A)** were obtained from the AGAR dataset (Majchrowska et al., 2025), licensed under CC BY-NC 2.0. Source images in **(B)** were obtained from the ADBC dataset (Makrai et al., 2023), licensed under CC BY 4.0.

Similarly, ICC also faces challenging scenarios in the ADBC dataset (Figure 6B). In addition to overlapping colonies, this dataset presents more difficult conditions, such as reflections, uneven illumination, and colonies with appearances that differ significantly from those in the training dataset, making detection more challenging. Detection errors involving overlapping colonies can often be corrected through user guidance. Colonies affected by reflection or uneven illumination can also often be correctly detected with user guidance, when the colony boundaries and key morphological features remain sufficiently discernible. However, when colonies have highly atypical appearances, it becomes difficult for ICC to distinguish individual colonies accurately.

## 5 Conclusion

The results demonstrate that the Interactive Colony Counter (ICC) provides a lightweight, user-guided framework for class-agnostic colony detection and annotation. By incorporating user-provided bounding boxes into an iterative human-in-the-loop process, ICC progressively refines its predictions and helps mitigate the limited generalization of fully automated colony-counting models across diverse colony types and imaging conditions. The stronger performance of the bounding-box interaction strategy than the key-point variant further indicates that bounding boxes provide more informative spatial and contextual cues for colony detection.

Across the higher- and lower-resolution AGAR subsets, ICC achieved competitive detection and counting performance, while evaluation on the ADBC dataset provided an external assessment under different acquisition conditions and included species not represented in the training data. The overall improvement with successive interactions, together with the lightweight design for direct processing of high-resolution whole-plate images, highlights the effectiveness and practicality of the proposed framework. Its integration into a semi-automatic annotation tool further enabled the construction of the *B. lactis*-Colony dataset, demonstrating the feasibility of ICC-assisted microbial image annotation and dataset development. The manual corrections were not independently audited, the target and non-target categories were assigned primarily based on colony morphology, and real-user annotation accuracy and workload will be evaluated in future work. In addition, retraining on the TXL-PBC dataset indicated that the proposed architecture can be transferred to another cell-level detection task, although this represents task transfer rather than direct cross-domain generalization.

Despite these advantages, several limitations remain. First, ICC still relies on user guidance when multiple colony types coexist in the same image, particularly when their visual characteristics are difficult to distinguish. Second, the use of grayscale input improves robustness to variations in image acquisition but inevitably discards potentially informative color cues. Third, colonies with indistinct boundaries or highly atypical appearances remain challenging to resolve, and user interaction may provide only limited improvement in such cases.

Further studies could explore more effective use of color and contextual information, reduced reliance on user interaction, and broader evaluation across microbial datasets and resource-constrained platforms. Evaluation using clinical samples would also help assess the practical utility of ICC in real-world settings. Together, these efforts are expected to improve the robustness and usability of ICC and further support its application to practical microbial colony detection, counting, and image annotation workflows.

## Data Availability

The raw data supporting the conclusions of this article will be made available by the authors, without undue reservation.

## References

[ref1] Albaradei S. A. Napolitano F. Uludag M. Thafar M. Napolitano S. Essack M. . (2020). Automated counting of colony forming units using deep transfer learning from a model for congested scenes analysis. IEEE Access 8, 164340–164346. doi: 10.1109/ACCESS.2020.3021656

[ref2] Beznik T. Smyth P. de Lannoy G. Lee J. A. (2022). Deep learning to detect bacterial colonies for the production of vaccines. Neurocomputing 470, 427–431. doi: 10.1016/j.neucom.2021.04.130

[ref3] Brugger S. D. Baumberger C. Jost M. Jenni W. Brugger U. Mühlemann K. (2012). Automated counting of bacterial colony forming units on agar plates. PLoS One 7:e33695. doi: 10.1371/journal.pone.0033695, 22448267 PMC3308999

[ref4] Cai Z. Vasconcelos N. Cascade r-cnn: delving into high quality object detection Proceedings of the IEEE Conference on Computer vision and Pattern Recognition (2018). 6154–6162

[ref5] Chen X. Zhao Z. Zhang Y. Duan M. Qi D. Zhao H. Focalclick: towards practical interactive image segmentation Proceedings of the IEEE/CVF Conference on Computer Vision and Pattern Recognition (2022). 1300–1309

[ref6] Choi D. Cho W. Kim K. Choo J. idet3d: towards efficient interactive object detection for lidar point clouds, Proceedings of the AAAI Conference on Artificial Intelligence. (2024). 1335–1343

[ref7] Clarke M. L. Burton R. L. Hill A. N. Litorja M. Nahm M. H. Hwang J. (2010). Low-cost, high-throughput, automated counting of bacterial colonies. Cytometry 77, 790–797. doi: 10.1002/cyto.a.20864, 20140968 PMC2909336

[ref8] Deng J. Dong W. Socher R. Li L.-J. Li K. Fei-Fei L. Imagenet: a large-scale hierarchical image database 2009 IEEE Conference on Computer vision and Pattern Recognition. Miami, FL, USA: IEEE, (2009). 248–255

[ref9] Ding H. Cohen S. Price B. Jiang X. Phraseclick: toward achieving flexible interactive segmentation by phrase and click. European Conference on Computer Vision. (2020). 417–435

[ref10] Everingham M. Van Gool L. Williams C. K. Winn J. Zisserman A. (2010). The pascal visual object classes (VOC) challenge. Int. J. Comput. Vis. 88, 303–338. doi: 10.1007/s11263-009-0275-4

[ref11] Ferrari A. Lombardi S. Signoroni A. Bacterial colony counting by convolutional neural networks. 2015 37th Annual International Conference of the IEEE Engineering in medicine and Biology Society (EMBC). (2015). 7458–7461 10.1109/EMBC.2015.7320116 26738016

[ref12] Gan L. Li X. Wang X. (2025). A curated and re-annotated peripheral blood cell dataset integrating four public resources. Sci. Data 12:1694. doi: 10.1038/s41597-025-05980-z, 41145591 PMC12559723

[ref13] Garcia-Armesto M. Prieto M. García-López M. Otero A. Moreno B. (1993). Modern microbiological methods for foods: colony count and direct count methods. A review. Microbiologia 9, 1–13. 8397961

[ref14] He K. Zhang X. Ren S. Sun J. Deep residual learning for image recognition Proceedings of the IEEE Conference on Computer vision and Pattern Recognition, Los Alamitos, CA. (2016). 770–778

[ref15] Hsiao W. W.-W. Qureshi S. A. Aman H. Chang S.-W. Saravanan A. Lam X. M. (2025). Artificial intelligence-enabled predictive system for *Escherichia coli* colony counting using patch-based supervised cytometry regression: a technical framework. Microchem. J. 212:113206. doi: 10.1016/j.microc.2025.113206

[ref16] Jocher G. (2020). Ultralytics YOLOv5. Geneva, Switzerland: Ultralytics.

[ref17] Jocher G. Chaurasia A. Qiu J. (2023). Ultralytics YOLOv8. San Francisco, CA, USA: Ultralytics.

[ref18] Jocher G. Qiu J. Liu M. Lyu S. Akyon F.C. Kalfaoglu M.E. Ultralytics YOLO26: Unified Real-Time End-to-End Vision Models, Ithaca, NY, USA: arXiv.org, (2026), doi: 10.48550/arXiv.2606.03748

[ref19] Kirillov A. Mintun E. Ravi N. Mao H. Rolland C. Gustafson L. . Segment anything Proceedings of the IEEE/CVF International Conference on Computer vision, Los Alamitos, CA (2023). 4015–4026

[ref20] Lee C. Park S. Song H. Ryu J. Kim S. Kim H. . Interactive multi-class tiny-object detection. Proceedings of the IEEE/CVF Conference on Computer Vision and Pattern Recognition. (2022). 14136–14145

[ref21] Li C. Li L. Jiang H. Weng K. Geng Y. Li L. . (2022). YOLOv6: a single-stage object detection framework for industrial applications. arXiv. doi: 10.48550/arXiv.2209.02976

[ref22] Li Y. Zhang X. Chen D. Csrnet: dilated convolutional neural networks for understanding the highly congested scenes. Proceedings of the IEEE Conference on Computer vision and Pattern Recognition. (2018). 1091–1100

[ref23] Lin T.-Y. Dollár P. Girshick R. He K. Hariharan B. Belongie S. Feature pyramid networks for object detection Proceedings of the IEEE Conference on Computer vision and Pattern Recognition, Los Alamitos, CA. (2017). 2117–2125

[ref24] Lin Z. Zhang Z. Chen L.-Z. Cheng M.-M. Lu S.-P. Interactive image segmentation with first click attention Proceedings of the IEEE/CVF Conference on Computer vision and Pattern Recognition. (2020). 13339–13348

[ref25] Liu Q. Xu Z. Bertasius G. Niethammer M. Simpleclick: interactive image segmentation with simple vision transformers Proceedings of the IEEE/CVF International Conference on Computer Vision. (2023). 22290–22300 10.1109/iccv51070.2023.02037 PMC11378330 39247160

[ref26] Majchrowska J. P. S. Guła G. Bonus T. Hanas A. Loch A. Pawlak A. . (2025). Assessing microbial colony counting: a deep learning approach with the AGAR image dataset. Neurocomputing 630:129654. doi: 10.1016/j.neucom.2025.129654

[ref27] Makrai L. Fodróczy B. Nagy S. Á. Czeiszing P. Csabai I. Szita G. . (2023). Annotated dataset for deep-learning-based bacterial colony detection. Sci. Data 10:497. doi: 10.1038/s41597-023-02404-8, 37507412 PMC10382471

[ref28] Minaee S. Boykov Y. Porikli F. Plaza A. Kehtarnavaz N. Terzopoulos D. (2022). Image segmentation using deep learning: a survey. IEEE Trans. Pattern Anal. Mach. Intell. 44, 3523–3542. doi: 10.1109/tpami.2021.3059968 33596172

[ref29] Morelli R. Clissa L. Amici R. Cerri M. Hitrec T. Luppi M. . (2021). Automating cell counting in fluorescent microscopy through deep learning with c-ResUnet. Sci. Rep. 11:22920. doi: 10.1038/s41598-021-01929-5, 34824294 PMC8617067

[ref30] Nunn C. J. Klyshko E. Goyal S. (2023). petiteFinder: an automated computer vision tool to compute petite colony frequencies in baker’s yeast. BMC Bioinformatics 24:50. doi: 10.1186/s12859-023-05168-5, 36793007 PMC9930278

[ref31] Ren S. He K. Girshick R. Sun J. (2016). Faster R-CNN: towards real-time object detection with region proposal networks. IEEE Trans. Pattern Anal. Mach. Intell. 39, 1137–1149. doi: 10.1109/TPAMI.2016.2577031 27295650

[ref32] Rodrigues P. M. Luís J. Tavaria F. K. (2022). Image analysis semi-automatic system for colony-forming-unit counting. Bioengineering 9:271. doi: 10.3390/bioengineering9070271, 35877322 PMC9312004

[ref33] Sannidhan M. S. Martis J. E. Krivic S. Sudeepa K. B. Nazareth P. (2024). A swarm-optimized microbial colony counter. Expert. Syst. 41:e13510. doi: 10.1111/exsy.13510

[ref34] Selvaraju R. R. Cogswell M. Das A. Vedantam R. Parikh D. Batra D. Grad-cam: visual explanations from deep networks via gradient-based localization. Proceedings of the IEEE International Conference on Computer vision. (2017). 618–626

[ref35] Sieuwerts S. De Bok F. A. Mols E. De Vos W. M. van Hylckama Vlieg J. (2008). A simple and fast method for determining colony forming units. Lett. Appl. Microbiol. 47, 275–278. doi: 10.1111/j.1472-765X.2008.02417.x 18778376

[ref36] Sofiiuk K. Petrov I. A. Konushin A. Reviving iterative training with mask guidance for interactive segmentation 2022 IEEE International Conference on Image Processing (ICIP). (2022). 3141–3145

[ref37] Terven J. Córdova-Esparza D.-M. Romero-González J.-A. (2023). A comprehensive review of YOLO architectures in computer vision: from YOLOv1 to YOLOv8 and YOLO-NAS. Mach. Learn. Knowl. Extr. 5, 1680–1716. doi: 10.3390/make5040083

[ref38] Wang C.-Y. Bochkovskiy A. Liao H.-Y.M. YOLOv7: trainable bag-of-freebies sets new state-of-the-art for real-time object detectors, Proceedings of the IEEE/CVF Conference on Computer vision and Pattern Recognition, Los Alamitos, CA. (2023) 7464–7475

[ref39] Wang G. Ye J. C. De Man B. (2020). Deep learning for tomographic image reconstruction. Nat. Mach. Intell. 2, 737–748. doi: 10.1038/s42256-020-00273-z

[ref40] Whipp J. Dong A. Yolo-based deep learning to automated bacterial colony counting 2022 IEEE Eighth International Conference on Multimedia Big Data (BigMM). (2022). 120–124

[ref41] Woo S. Park J. Lee J.-Y. Kweon I.S. Cbam: convolutional block attention module. Proceedings of the European Conference on Computer vision (ECCV). (2018). 3–19

[ref42] Zhang L. (2022). Machine learning for enumeration of cell colony forming units. Visual Comput Industry Biomed Art 5:26. doi: 10.1186/s42492-022-00122-3, 36334176 PMC9637067

[ref43] Zhang C. Chen W.-B. Liu W.-L. Chen C.-B. An automated bacterial colony counting system 2008 IEEE International Conference on sensor Networks, Ubiquitous, and Trustworthy Computing (SUTC 2008). (2008) 233–240

[ref44] Zhang Y. Chen J. Ma X. Wang G. Bhatti U. A. Huang M. (2024). Interactive medical image annotation using improved attention U-net with compound geodesic distance. Expert Syst. Appl. 237:121282. doi: 10.1016/j.eswa.2023.121282

[ref45] Zhang J. Li C. Rahaman M. M. Yao Y. Ma P. Zhang J. . (2022). A comprehensive review of image analysis methods for microorganism counting: from classical image processing to deep learning approaches. Artif. Intell. Rev. 55, 2875–2944. doi: 10.1007/s10462-021-10082-4, 34602697 PMC8478609

[ref46] Zheng Z. Wang P. Liu W. Li J. Ye R. Ren D. Distance-IoU loss: faster and better learning for bounding box regression. Proceedings of the AAAI Conference on artificial Intelligence. (2020) 12993–13000

[ref47] Zou Z. Chen K. Shi Z. Guo Y. Ye J. (2023). Object detection in 20 years: a survey. Proc. IEEE 111, 257–276. doi: 10.1109/jproc.2023.3238524

