# Supplementary material for: An interactive class-agnostic detection framework for generalizable colony counting
Source: Front Microbiol. 2026 Oct 21;17:1967912. doi: 10.3389/fmicb.2026.1967912 (PMC13761786; doi:10.3389/fmicb.2026.1967912)
Supplement: Supplementary file 1 [file Supplementary_file_1.DOCX]

**Section S1: Tables and Figures**

*Table S1. Detection result on AGAR higher-resolution subset*

| Species | IoU thresh | Faster R-CNN | Cascade R-CNN | ICC-Kpt | | | | | | | | | | | ICC-Bbox | | | | | | | | | | |
| --- | --- | --- | --- | --- | --- | --- | --- | --- | --- | --- | --- | --- | --- | --- | --- | --- | --- | --- | --- | --- | --- | --- | --- | --- | --- |
|  |  | ResNet-50 | HRNet | Iter 0 | Iter 1 | Iter 2 | Iter 3 | Iter 4 | Iter 5 | Iter 6 | Iter 7 | Iter 8 | Iter 9 | Iter 10 | Iter 0 | Iter 1 | Iter 2 | Iter 3 | Iter 4 | Iter 5 | Iter 6 | Iter 7 | Iter 8 | Iter 9 | Iter 10 |
| *S.aureus* | 0.5 | 86.00% | **95.80%** | 90.92% | 91.52% | 91.89% | 92.01% | 92.05% | 92.06% | 92.85% | 92.13% | 92.85% | 92.86% | 92.14% | 92.21% | 93.84% | 94.31% | 94.63% | 94.87% | 95.02% | 95.08% | 95.19% | 95.30% | 95.37% | 95.38% |
|  | 0.55 | 85.90% | 93.80% | 88.43% | 88.93% | 89.25% | 89.34% | 90.09% | 89.45% | 90.17% | 90.17% | 90.13% | 90.08% | 90.06% | 90.91% | 92.53% | 93.01% | 93.45% | 93.71% | 93.85% | 93.89% | 94.01% | 94.08% | 94.15% | **94.08%** |
|  | 0.6 | 84.80% | 90.80% | 83.84% | 84.22% | 84.49% | 84.60% | 84.62% | 84.52% | 84.52% | 84.40% | 84.32% | 84.31% | 84.27% | 88.18% | 90.36% | 90.91% | 91.40% | 91.65% | 92.00% | 92.20% | 92.31% | 92.37% | 92.41% | **92.37%** |
|  | 0.65 | 80.90% | **89.70%** | 75.79% | 76.56% | 76.74% | 76.50% | 76.47% | 76.27% | 75.54% | 76.13% | 75.40% | 75.33% | 75.29% | 83.91% | 86.48% | 87.74% | 88.43% | 88.69% | 89.01% | 89.20% | 89.50% | 89.41% | 89.42% | 89.46% |
|  | 0.7 | 77.90% | 79.40% | 63.96% | 63.97% | 64.15% | 63.82% | 63.67% | 63.39% | 63.19% | 63.13% | 63.04% | 62.81% | 62.62% | 75.76% | 79.46% | 80.88% | 81.79% | 82.75% | 83.26% | 83.81% | 83.98% | 84.36% | 84.48% | **84.65%** |
|  | 0.75 | 71.10% | 74.40% | 47.88% | 47.48% | 47.19% | 46.97% | 46.77% | 46.61% | 46.44% | 46.25% | 46.09% | 45.35% | 45.10% | 62.62% | 66.10% | 69.13% | 71.19% | 73.15% | 74.11% | 74.69% | 75.25% | 75.91% | 75.99% | **76.35%** |
|  | 0.8 | 58.40% | **64.50%** | 28.89% | 28.56% | 28.26% | 27.92% | 27.14% | 26.96% | 26.84% | 26.65% | 26.50% | 26.31% | 26.14% | 45.25% | 48.01% | 50.65% | 52.76% | 54.96% | 56.01% | 57.28% | 58.53% | 59.76% | 60.43% | 60.98% |
|  | 0.85 | 37.40% | **46.50%** | 11.74% | 11.65% | 11.47% | 11.29% | 11.16% | 10.91% | 10.85% | 10.69% | 10.67% | 10.62% | 10.48% | 23.24% | 25.71% | 27.49% | 28.86% | 31.14% | 32.54% | 34.06% | 35.29% | 36.34% | 37.37% | 38.22% |
|  | 0.9 | 14.80% | **18.00%** | 2.13% | 2.10% | 1.94% | 1.92% | 1.91% | 1.92% | 1.90% | 1.87% | 1.83% | 1.81% | 1.79% | 5.20% | 6.44% | 7.46% | 8.29% | 9.13% | 9.61% | 10.38% | 11.13% | 12.00% | 12.44% | 12.97% |
|  | 0.95 | 0.60% | **2.90%** | 0.03% | 0.03% | 0.02% | 0.03% | 0.03% | 0.03% | 0.02% | 0.02% | 0.02% | 0.02% | 0.02% | 0.12% | 0.22% | 0.27% | 0.32% | 0.35% | 0.41% | 0.49% | 0.50% | 0.54% | 0.57% | 0.63% |
|  | 0.5:0.95 | 59.78% | **65.58%** | 49.36% | 49.50% | 49.54% | 49.44% | 49.39% | 49.21% | 49.23% | 49.14% | 49.09% | 48.95% | 48.79% | 56.74% | 58.92% | 60.18% | 61.11% | 62.04% | 62.58% | 63.11% | 63.57% | 64.01% | 64.26% | 64.51% |
| *B.subtilis* | 0.5 | 72.40% | 73.60% | 91.27% | 92.73% | 93.30% | 93.67% | 93.80% | 93.91% | 93.96% | 94.77% | 94.86% | 94.98% | 95.07% | 93.65% | 94.51% | 95.12% | 95.61% | 95.69% | 95.82% | 95.96% | 96.08% | 96.15% | 96.32% | **96.39%** |
|  | 0.55 | 69.50% | 70.80% | 89.22% | 90.89% | 91.54% | 92.01% | 92.23% | 93.24% | 93.38% | 93.55% | 93.61% | 93.75% | 93.78% | 92.06% | 93.14% | 93.75% | 94.08% | 94.44% | 94.58% | 94.96% | 95.17% | 95.26% | 95.40% | **95.60%** |
|  | 0.6 | 64.60% | 65.70% | 86.09% | 87.56% | 88.90% | 89.65% | 89.97% | 90.16% | 90.39% | 90.63% | 90.66% | 91.51% | 91.66% | 89.89% | 91.01% | 91.68% | 92.22% | 92.60% | 92.93% | 93.08% | 93.36% | 93.82% | 93.89% | **93.99%** |
|  | 0.65 | 56.90% | 59.10% | 82.48% | 83.75% | 84.29% | 85.87% | 86.33% | 86.60% | 86.81% | 87.06% | 87.97% | 88.18% | 88.36% | 87.17% | 88.31% | 89.28% | 89.81% | 90.19% | 90.56% | 90.84% | 91.12% | 91.30% | 91.51% | **91.79%** |
|  | 0.7 | 48.90% | 50.90% | 75.58% | 76.77% | 77.27% | 78.69% | 79.40% | 80.04% | 80.60% | 81.74% | 81.99% | 82.10% | 82.38% | 82.02% | 83.50% | 84.39% | 85.03% | 85.55% | 86.14% | 86.47% | 86.86% | 87.27% | 87.46% | **87.66%** |
|  | 0.75 | 39.80% | 41.60% | 64.17% | 65.32% | 67.00% | 67.84% | 69.38% | 70.11% | 70.74% | 71.78% | 72.14% | 72.46% | 72.86% | 72.82% | 74.42% | 75.41% | 76.19% | 76.87% | 77.08% | 77.69% | 78.29% | 78.74% | 79.18% | **79.45%** |
|  | 0.8 | 30.00% | 31.20% | 46.57% | 46.93% | 48.06% | 48.81% | 50.80% | 52.12% | 53.59% | 54.11% | 54.59% | 55.18% | 55.34% | 57.82% | 59.84% | 61.07% | 61.79% | 62.67% | 63.27% | 64.07% | 64.63% | 64.92% | 65.12% | **65.56%** |
|  | 0.85 | 17.50% | 19.20% | 25.14% | 24.26% | 24.39% | 25.51% | 26.41% | 28.47% | 29.42% | 30.25% | 30.71% | 30.90% | 31.64% | 35.79% | 37.45% | 38.38% | 39.03% | 40.11% | 41.09% | 41.98% | 42.72% | 43.24% | 44.18% | **44.68%** |
|  | 0.9 | 6.30% | 7.60% | 6.54% | 6.36% | 6.14% | 6.80% | 7.01% | 7.68% | 7.77% | 7.96% | 7.97% | 8.47% | 8.58% | 11.27% | 13.26% | 14.00% | 15.08% | 15.72% | 16.70% | 17.19% | 18.13% | 18.86% | 19.60% | **20.14%** |
|  | 0.95 | 0.40% | 1.30% | 0.12% | 0.12% | 0.12% | 0.12% | 0.12% | 0.13% | 0.13% | 0.13% | 0.19% | 0.20% | 0.20% | 0.38% | 0.64% | 0.91% | 1.11% | 1.27% | 1.39% | 1.49% | 1.76% | 2.00% | 2.20% | **2.50%** |
|  | 0.5:0.95 | 40.63% | 42.10% | 56.72% | 57.47% | 58.10% | 58.90% | 59.55% | 60.25% | 60.68% | 61.20% | 61.47% | 61.77% | 61.99% | 62.29% | 63.61% | 64.40% | 64.99% | 65.51% | 65.96% | 66.37% | 66.81% | 67.16% | 67.49% | **67.78%** |
| *P.aeruginosa* | 0.5 | 69.90% | 71.70% | 83.50% | 86.64% | 88.02% | 89.16% | 89.42% | 90.44% | 90.68% | 90.90% | 90.97% | 91.82% | 91.97% | 86.45% | 89.60% | 90.85% | 91.66% | 92.38% | 92.88% | 93.16% | 93.35% | 93.57% | 93.81% | **94.00%** |
|  | 0.55 | 67.40% | 70.70% | 80.95% | 84.30% | 86.26% | 87.56% | 87.82% | 88.20% | 88.95% | 89.05% | 89.28% | 89.42% | 89.52% | 84.56% | 87.45% | 88.88% | 89.91% | 90.54% | 91.11% | 91.66% | 91.90% | 92.12% | 92.24% | **92.47%** |
|  | 0.6 | 65.60% | 68.90% | 77.39% | 80.87% | 82.85% | 83.45% | 84.65% | 85.07% | 85.26% | 85.90% | 86.06% | 86.21% | 86.37% | 81.66% | 85.01% | 86.50% | 87.27% | 87.93% | 88.33% | 88.68% | 89.08% | 89.35% | 89.63% | **89.87%** |
|  | 0.65 | 62.40% | 65.50% | 72.10% | 74.78% | 76.56% | 78.14% | 78.61% | 79.89% | 80.24% | 80.62% | 80.87% | 81.71% | 81.84% | 77.67% | 80.96% | 82.63% | 83.58% | 84.29% | 84.78% | 85.14% | 85.48% | 85.77% | 85.99% | **86.25%** |
|  | 0.7 | 57.90% | 61.50% | 63.00% | 66.55% | 67.72% | 69.39% | 70.82% | 71.29% | 71.78% | 72.88% | 73.14% | 73.46% | 73.55% | 70.65% | 74.38% | 75.85% | 76.90% | 77.63% | 78.45% | 78.76% | 79.04% | 79.43% | 79.74% | **79.99%** |
|  | 0.75 | 50.90% | 53.80% | 50.26% | 53.53% | 55.10% | 56.81% | 57.54% | 59.07% | 59.43% | 60.61% | 60.98% | 62.05% | 62.29% | 60.89% | 64.33% | 66.29% | 67.47% | 68.31% | 68.86% | 69.45% | 69.95% | 70.47% | 70.96% | **71.31%** |
|  | 0.8 | 38.00% | 42.70% | 34.78% | 36.93% | 38.85% | 40.63% | 41.56% | 42.86% | 43.44% | 44.47% | 44.99% | 45.84% | 46.33% | 46.28% | 50.57% | 52.17% | 53.17% | 54.14% | 54.84% | 55.65% | 56.18% | 56.82% | 57.43% | **57.94%** |
|  | 0.85 | 23.30% | 25.50% | 17.93% | 18.39% | 19.37% | 20.32% | 21.30% | 22.40% | 22.86% | 23.65% | 24.01% | 24.11% | 24.82% | 27.71% | 31.09% | 32.74% | 34.19% | 34.97% | 36.01% | 36.80% | 37.48% | 38.00% | 38.52% | **39.09%** |
|  | 0.9 | 7.90% | 8.30% | 4.15% | 4.19% | 4.40% | 4.68% | 5.03% | 5.13% | 5.42% | 5.69% | 5.75% | 5.81% | 5.90% | 9.34% | 11.82% | 12.95% | 13.69% | 14.58% | 15.55% | 16.00% | 16.60% | 17.20% | 17.65% | **18.27%** |
|  | 0.95 | 0.70% | 0.80% | 0.07% | 0.07% | 0.11% | 0.07% | 0.08% | 0.11% | 0.12% | 0.12% | 0.12% | 0.13% | 0.13% | 0.29% | 0.75% | 1.03% | 1.20% | 1.44% | 1.70% | 1.84% | 1.97% | 2.11% | 2.34% | **2.42%** |
|  | 0.5:0.95 | 44.40% | 46.94% | 48.41% | 50.63% | 51.92% | 53.02% | 53.68% | 54.45% | 54.82% | 55.39% | 55.62% | 56.06% | 56.27% | 54.55% | 57.60% | 58.99% | 59.90% | 60.62% | 61.25% | 61.71% | 62.10% | 62.49% | 62.83% | **63.16%** |
| *E.coli* | 0.5 | 73.10% | 74.60% | 88.24% | 90.27% | 92.12% | 92.46% | 92.53% | 92.58% | 92.65% | 92.70% | 92.70% | 93.49% | 93.58% | 90.50% | 92.85% | 93.69% | 94.01% | 94.29% | 94.52% | 94.75% | 95.05% | 95.18% | 95.27% | **95.31%** |
|  | 0.55 | 68.90% | 71.50% | 86.25% | 88.38% | 90.29% | 90.83% | 90.97% | 91.06% | 91.04% | 91.14% | 91.21% | 91.17% | 91.23% | 88.89% | 91.40% | 92.18% | 92.50% | 92.74% | 93.06% | 93.32% | 93.50% | 93.62% | 93.80% | **93.90%** |
|  | 0.6 | 64.00% | 66.60% | 83.10% | 86.25% | 88.16% | 88.70% | 89.04% | 89.25% | 89.25% | 89.35% | 89.41% | 89.46% | 89.48% | 86.23% | 88.78% | 89.79% | 90.18% | 90.51% | 90.88% | 91.03% | 91.22% | 91.31% | 91.39% | **91.45%** |
|  | 0.65 | 56.60% | 59.30% | 78.12% | 81.28% | 83.56% | 84.35% | 84.68% | 84.91% | 84.97% | 85.24% | 85.36% | 85.42% | 85.44% | 83.01% | 85.69% | 86.82% | 87.38% | 87.68% | 87.97% | 88.18% | 88.39% | 88.54% | 88.70% | **88.74%** |
|  | 0.7 | 47.90% | 51.20% | 71.26% | 73.65% | 76.94% | 78.01% | 78.34% | 78.72% | 79.03% | 80.07% | 80.29% | 80.34% | 80.47% | 77.60% | 80.72% | 81.85% | 82.33% | 82.68% | 82.99% | 83.23% | 83.36% | 83.66% | 83.83% | **84.00%** |
|  | 0.75 | 39.00% | 41.70% | 60.54% | 62.31% | 64.83% | 66.85% | 67.79% | 68.19% | 69.22% | 69.62% | 69.81% | 70.08% | 70.20% | 68.87% | 71.91% | 73.50% | 74.34% | 74.70% | 75.02% | 75.24% | 75.59% | 75.84% | 76.01% | **76.19%** |
|  | 0.8 | 30.20% | 33.10% | 45.44% | 46.63% | 48.59% | 49.87% | 51.28% | 52.03% | 53.24% | 53.78% | 54.13% | 54.92% | 55.10% | 55.13% | 58.33% | 59.84% | 60.74% | 61.26% | 61.68% | 62.22% | 62.54% | 62.71% | 63.01% | **63.31%** |
|  | 0.85 | 19.40% | 22.30% | 24.90% | 25.59% | 26.42% | 27.60% | 28.24% | 28.72% | 29.50% | 29.84% | 30.20% | 30.87% | 31.12% | 35.52% | 38.59% | 40.04% | 40.63% | 41.15% | 41.67% | 42.11% | 42.44% | 42.63% | 43.04% | **43.42%** |
|  | 0.9 | 7.40% | 10.20% | 6.56% | 6.57% | 6.81% | 6.94% | 7.34% | 7.50% | 7.67% | 7.86% | 8.18% | 8.27% | 8.36% | 12.39% | 14.53% | 15.55% | 16.02% | 16.50% | 16.87% | 17.34% | 17.62% | 17.86% | 18.16% | **18.60%** |
|  | 0.95 | 0.50% | 0.80% | 0.21% | 0.19% | 0.19% | 0.19% | 0.20% | 0.21% | 0.21% | 0.22% | 0.22% | 0.22% | 0.23% | 0.44% | 0.89% | 1.15% | 1.35% | 1.50% | 1.67% | 1.72% | 1.80% | 1.87% | 2.02% | **2.07%** |
|  | 0.5:0.95 | 40.70% | 43.13% | 54.46% | 56.11% | 57.79% | 58.58% | 59.04% | 59.32% | 59.68% | 59.98% | 60.15% | 60.42% | 60.52% | 59.86% | 62.37% | 63.44% | 63.95% | 64.30% | 64.63% | 64.91% | 65.15% | 65.32% | 65.52% | **65.70%** |
| *C.albicans* | 0.5 | 82.00% | 82.10% | 71.15% | 73.47% | 74.69% | 75.03% | 75.88% | 76.03% | 76.76% | 76.83% | 76.94% | 76.97% | 77.65% | 77.78% | 81.59% | 83.53% | 84.77% | 85.88% | 86.57% | 87.06% | 87.32% | 87.49% | 87.78% | **87.96%** |
|  | 0.55 | 82.00% | 82.00% | 66.57% | 67.86% | 68.93% | 69.14% | 69.88% | 69.94% | 70.02% | 70.59% | 70.71% | 70.81% | 70.88% | 74.85% | 78.98% | 80.75% | 81.85% | 82.72% | 83.46% | 83.83% | 84.45% | 84.76% | 84.97% | **85.02%** |
|  | 0.6 | 80.80% | 80.90% | 59.46% | 60.62% | 60.91% | 61.70% | 61.85% | 62.46% | 62.37% | 62.39% | 62.45% | 62.51% | 62.50% | 69.92% | 74.22% | 75.98% | 77.42% | 78.28% | 79.09% | 79.75% | 80.17% | 80.58% | 80.91% | **81.17%** |
|  | 0.65 | 79.60% | **79.80%** | 49.53% | 50.46% | 50.77% | 51.04% | 51.00% | 51.50% | 51.49% | 51.57% | 51.63% | 52.16% | 52.07% | 62.31% | 66.90% | 69.42% | 70.83% | 72.06% | 72.46% | 73.06% | 73.91% | 74.31% | 74.70% | 75.02% |
|  | 0.7 | 77.10% | **78.40%** | 37.27% | 37.37% | 37.53% | 37.80% | 38.12% | 38.24% | 38.09% | 38.16% | 38.15% | 38.16% | 38.18% | 51.60% | 57.27% | 59.73% | 60.98% | 61.96% | 62.64% | 63.09% | 63.47% | 64.12% | 64.23% | 64.54% |
|  | 0.75 | 73.20% | **74.80%** | 23.64% | 24.37% | 24.25% | 24.42% | 24.40% | 24.41% | 24.19% | 24.18% | 24.16% | 24.19% | 24.27% | 37.52% | 43.13% | 45.75% | 47.61% | 48.49% | 49.60% | 50.27% | 50.91% | 51.33% | 51.69% | 51.82% |
|  | 0.8 | 65.50% | **68.10%** | 12.78% | 12.99% | 13.09% | 12.99% | 12.91% | 12.82% | 12.71% | 12.67% | 12.69% | 12.70% | 12.70% | 23.06% | 28.12% | 30.34% | 31.95% | 33.19% | 33.94% | 34.73% | 35.11% | 35.44% | 35.75% | 35.98% |
|  | 0.85 | 49.40% | **52.40%** | 5.11% | 5.16% | 5.16% | 5.01% | 4.96% | 4.95% | 4.85% | 4.79% | 4.82% | 4.75% | 4.72% | 9.82% | 13.49% | 15.42% | 16.49% | 17.71% | 18.53% | 18.95% | 19.39% | 19.63% | 19.82% | 19.96% |
|  | 0.9 | 19.20% | **22.10%** | 0.70% | 0.69% | 0.73% | 0.73% | 0.72% | 0.72% | 0.69% | 0.66% | 0.66% | 0.65% | 0.66% | 1.72% | 3.24% | 4.23% | 4.98% | 5.24% | 5.69% | 6.03% | 6.15% | 6.26% | 6.41% | 6.50% |
|  | 0.95 | 1.80% | **2.70%** | 0.00% | 0.00% | 0.00% | 0.00% | 0.00% | 0.00% | 0.00% | 0.00% | 0.00% | 0.00% | 0.00% | 0.03% | 0.08% | 0.15% | 0.15% | 0.16% | 0.19% | 0.20% | 0.21% | 0.22% | 0.22% | 0.24% |
|  | 0.5:0.95 | 61.06% | **62.33%** | 32.62% | 33.30% | 33.61% | 33.79% | 33.97% | 34.11% | 34.12% | 34.18% | 34.22% | 34.29% | 34.36% | 40.86% | 44.70% | 46.53% | 47.70% | 48.57% | 49.22% | 49.70% | 50.11% | 50.41% | 50.65% | 50.82% |

*Table S2. Detection result on AGAR lower-resolution subset*

| Species | IoU thresh | Faster R-CNN | Cascade R-CNN | ICC-Kpt | | | | | | | | | | | ICC-Bbox | | | | | | | | | | |
| --- | --- | --- | --- | --- | --- | --- | --- | --- | --- | --- | --- | --- | --- | --- | --- | --- | --- | --- | --- | --- | --- | --- | --- | --- | --- |
|  |  | ResNet-50 | HRNet | Iter 0 | Iter 1 | Iter 2 | Iter 3 | Iter 4 | Iter 5 | Iter 6 | Iter 7 | Iter 8 | Iter 9 | Iter 10 | Iter 0 | Iter 1 | Iter 2 | Iter 3 | Iter 4 | Iter 5 | Iter 6 | Iter 7 | Iter 8 | Iter 9 | Iter 10 |
| *S.aureus* | 0.5 | - | - | 88.55% | 91.35% | 92.46% | 92.74% | 92.92% | 92.96% | 92.92% | 92.92% | 92.98% | 92.98% | 93.01% | 86.43% | 91.14% | 92.96% | 93.91% | 94.48% | 94.89% | 95.25% | 95.44% | 95.61% | **95.62%** | 95.62% |
|  | 0.55 | - | - | 86.24% | 88.97% | 89.54% | 89.70% | 89.83% | 90.63% | 89.86% | 89.81% | 89.83% | 89.82% | 89.80% | 84.64% | 88.86% | 90.59% | 91.51% | 92.58% | 93.05% | 93.38% | 93.90% | 94.20% | 94.31% | **94.34%** |
|  | 0.6 | - | - | 82.96% | 84.41% | 85.44% | 85.62% | 85.82% | 85.89% | 85.92% | 85.83% | 85.76% | 85.79% | 85.75% | 81.63% | 85.84% | 88.06% | 89.00% | 90.13% | 90.83% | 91.09% | 91.27% | 91.48% | 91.54% | **91.79%** |
|  | 0.65 | - | - | 78.59% | 79.70% | 79.94% | 79.99% | 80.02% | 79.98% | 79.99% | 79.85% | 79.77% | 79.74% | 79.73% | 77.27% | 81.60% | 83.26% | 84.72% | 85.60% | 86.21% | 86.71% | 87.43% | 87.98% | 88.47% | **88.68%** |
|  | 0.7 | - | - | 72.31% | 73.14% | 73.02% | 73.05% | 72.71% | 72.59% | 72.40% | 72.33% | 72.38% | 72.31% | 72.22% | 71.20% | 74.26% | 76.29% | 77.89% | 78.90% | 79.82% | 81.00% | 81.51% | 82.22% | 82.56% | **82.92%** |
|  | 0.75 | - | - | 62.45% | 62.70% | 62.68% | 62.76% | 62.29% | 62.05% | 61.47% | 61.21% | 61.11% | 61.00% | 60.92% | 60.99% | 64.52% | 66.54% | 67.96% | 69.53% | 70.48% | 71.57% | 72.37% | 72.74% | 73.36% | **73.66%** |
|  | 0.8 | - | - | 46.82% | 47.08% | 47.39% | 47.06% | 46.49% | 45.76% | 45.51% | 45.40% | 45.37% | 45.30% | 45.37% | 45.05% | 48.05% | 50.20% | 52.27% | 53.47% | 55.29% | 55.73% | 57.12% | 57.64% | 58.11% | **58.29%** |
|  | 0.85 | - | - | 25.18% | 25.01% | 25.50% | 25.03% | 24.75% | 24.70% | 24.72% | 24.64% | 24.52% | 24.57% | 24.22% | 23.98% | 26.99% | 29.02% | 31.11% | 32.74% | 34.08% | 34.57% | 35.05% | 36.32% | 36.54% | **37.52%** |
|  | 0.9 | - | - | 6.15% | 6.49% | 6.59% | 6.18% | 6.10% | 6.04% | 5.78% | 5.76% | 5.75% | 5.68% | 5.69% | 5.76% | 7.32% | 8.57% | 9.30% | 9.96% | 10.99% | 11.69% | 12.21% | 12.76% | 13.37% | **13.92%** |
|  | 0.95 | - | - | 0.09% | 0.10% | 0.10% | 0.10% | 0.10% | 0.10% | 0.08% | 0.08% | 0.08% | 0.08% | 0.09% | 0.20% | 0.26% | 0.29% | 0.31% | 0.33% | 0.34% | 0.42% | 0.47% | 0.53% | 0.55% | **0.58%** |
|  | 0.5:0.95 | 66.50% | **69.20%** | 54.93% | 55.90% | 56.27% | 56.22% | 56.10% | 56.07% | 55.87% | 55.78% | 55.76% | 55.73% | 55.68% | 53.71% | 56.88% | 58.58% | 59.80% | 60.77% | 61.60% | 62.14% | 62.68% | 63.15% | 63.44% | 63.73% |
| *B.subtilis* | 0.5 | - | - | 73.43% | 81.56% | 84.79% | 86.02% | 87.92% | 88.92% | 88.74% | 89.69% | 89.78% | 89.79% | 89.87% | 83.74% | 88.54% | 90.18% | 90.48% | 91.34% | 91.75% | 92.10% | 92.41% | 92.55% | 92.76% | **92.91%** |
|  | 0.55 | - | - | 71.90% | 79.76% | 83.34% | 85.15% | 86.11% | 86.86% | 86.96% | 87.76% | 87.76% | 87.69% | 88.56% | 82.59% | 87.14% | 88.36% | 89.41% | 89.80% | 90.46% | 90.47% | 90.87% | 91.24% | 91.55% | **91.67%** |
|  | 0.6 | - | - | 70.34% | 77.79% | 80.43% | 82.26% | 83.36% | 84.27% | 85.19% | 85.10% | 86.10% | 86.39% | 86.49% | 80.95% | 84.90% | 85.09% | 86.94% | 87.71% | 88.23% | 88.42% | 88.53% | 88.42% | 88.05% | **88.20%** |
|  | 0.65 | - | - | 67.22% | 74.84% | 78.15% | 77.87% | 80.62% | 81.27% | 82.32% | 82.32% | 82.49% | 82.12% | 83.09% | 78.11% | 82.51% | 83.30% | 83.39% | 83.76% | 84.76% | 85.16% | 85.47% | 85.70% | 85.47% | **85.81%** |
|  | 0.7 | - | - | 62.27% | 68.91% | 70.76% | 73.53% | 74.54% | 75.30% | 75.72% | 76.45% | 76.45% | 76.59% | 76.55% | 72.78% | 76.94% | 78.49% | 78.97% | 79.94% | 80.31% | 80.09% | 80.53% | 80.78% | 81.43% | **81.69%** |
|  | 0.75 | - | - | 55.43% | 60.02% | 61.53% | 62.27% | 63.08% | 63.54% | 64.82% | 64.82% | 65.07% | 66.09% | 66.25% | 61.12% | 66.73% | 67.33% | 66.55% | 66.69% | 67.57% | 68.10% | 68.27% | 69.28% | 69.51% | **70.73%** |
|  | 0.8 | - | - | 41.42% | 44.63% | 45.75% | 44.97% | 45.48% | 46.50% | 45.86% | 45.97% | 46.21% | 46.96% | 47.79% | 44.90% | 48.10% | 49.31% | 49.46% | 49.81% | 49.88% | 50.95% | 50.94% | 51.22% | 52.38% | **53.06%** |
|  | 0.85 | - | - | 20.31% | 19.68% | 18.84% | 18.56% | 18.09% | 17.83% | 18.58% | 18.96% | 19.15% | 19.91% | 20.17% | 21.41% | 22.21% | 24.13% | 25.41% | 26.73% | 26.80% | 27.75% | 28.58% | 29.12% | 29.42% | **30.25%** |
|  | 0.9 | - | - | 3.17% | 3.11% | 3.27% | 3.17% | 3.60% | 3.74% | 3.93% | 4.13% | 4.30% | 4.32% | 4.38% | 4.66% | 5.84% | 7.15% | 8.65% | 8.58% | 8.80% | 9.14% | 9.83% | 10.68% | 10.75% | **11.21%** |
|  | 0.95 | - | - | 0.03% | 0.02% | 0.02% | 0.02% | 0.05% | 0.05% | 0.06% | 0.05% | 0.06% | 0.06% | 0.06% | 0.05% | 0.79% | 1.00% | 1.01% | 1.27% | 1.45% | 1.74% | 2.05% | 2.15% | 2.20% | **2.36%** |
|  | 0.5:0.95 | 41.10% | 48.00% | 46.55% | 51.03% | 52.69% | 53.38% | 54.29% | 54.83% | 55.22% | 55.53% | 55.74% | 55.99% | 56.32% | 53.03% | 56.37% | 57.43% | 58.03% | 58.56% | 59.00% | 59.39% | 59.75% | 60.11% | 60.35% | **60.79%** |
| *P.aeruginosa* | 0.5 | - | - | 55.34% | 66.91% | 83.46% | 87.44% | 88.96% | 90.29% | 91.49% | 91.22% | 92.47% | 92.34% | 93.36% | 60.38% | 78.71% | 87.87% | 91.09% | 92.11% | 92.48% | 93.08% | 93.81% | 94.44% | 94.80% | **95.03%** |
|  | 0.55 | - | - | 54.14% | 65.62% | 81.73% | 85.50% | 86.91% | 87.84% | 88.79% | 89.06% | 89.03% | 89.93% | 90.27% | 59.08% | 78.44% | 87.45% | 89.95% | 90.78% | 91.45% | 91.99% | 92.48% | 92.89% | 93.56% | **93.93%** |
|  | 0.6 | - | - | 52.68% | 63.62% | 77.74% | 82.77% | 83.94% | 85.48% | 86.41% | 86.08% | 87.05% | 87.18% | 87.73% | 57.23% | 75.55% | 84.66% | 87.61% | 88.67% | 89.52% | 90.38% | 90.85% | 91.49% | 91.79% | **92.06%** |
|  | 0.65 | - | - | 50.91% | 59.97% | 74.14% | 78.96% | 80.11% | 81.42% | 81.78% | 82.81% | 82.61% | 83.07% | 82.99% | 54.22% | 71.75% | 81.08% | 84.09% | 85.28% | 86.15% | 86.84% | 87.19% | 88.12% | 88.56% | **89.04%** |
|  | 0.7 | - | - | 45.82% | 53.99% | 66.92% | 70.18% | 72.25% | 72.47% | 73.23% | 73.43% | 73.61% | 74.75% | 74.81% | 48.69% | 65.77% | 74.93% | 78.67% | 80.37% | 81.35% | 82.18% | 82.66% | 82.75% | 83.13% | **83.65%** |
|  | 0.75 | - | - | 37.99% | 45.14% | 56.36% | 60.16% | 61.16% | 62.02% | 61.74% | 61.95% | 63.01% | 63.25% | 63.02% | 40.58% | 57.68% | 65.58% | 68.68% | 69.94% | 71.22% | 72.30% | 72.86% | 73.35% | 73.90% | **74.12%** |
|  | 0.8 | - | - | 27.41% | 32.23% | 41.38% | 43.83% | 45.23% | 45.07% | 45.77% | 45.22% | 45.30% | 45.44% | 45.52% | 29.51% | 43.41% | 50.73% | 54.09% | 55.82% | 57.01% | 58.06% | 58.71% | 59.37% | 59.53% | **59.84%** |
|  | 0.85 | - | - | 15.49% | 17.80% | 23.13% | 25.55% | 25.63% | 26.32% | 26.35% | 26.68% | 26.65% | 26.82% | 26.94% | 16.57% | 26.67% | 32.90% | 35.90% | 37.85% | 39.00% | 39.85% | 40.79% | 41.12% | 41.58% | **42.06%** |
|  | 0.9 | - | - | 4.04% | 5.13% | 6.67% | 7.17% | 7.32% | 7.61% | 7.65% | 7.59% | 7.56% | 7.78% | 7.76% | 4.89% | 12.30% | 14.97% | 17.17% | 18.52% | 19.43% | 20.22% | 21.02% | 21.31% | 21.46% | **21.73%** |
|  | 0.95 | - | - | 0.09% | 0.15% | 0.20% | 0.19% | 0.15% | 0.14% | 0.14% | 0.14% | 0.14% | 0.14% | 0.14% | 0.14% | 2.27% | 3.09% | 3.50% | 4.09% | 4.50% | 4.81% | 4.91% | **5.01%** | 4.90% | 4.88% |
|  | 0.5:0.95 | 50.60% | 54.70% | 34.39% | 41.06% | 51.17% | 54.18% | 55.17% | 55.87% | 56.34% | 56.42% | 56.74% | 57.07% | 57.25% | 37.13% | 51.25% | 58.33% | 61.08% | 62.34% | 63.21% | 63.97% | 64.53% | 64.99% | 65.32% | **65.64%** |
| *E.coli* | 0.5 | - | - | 64.71% | 77.20% | 92.30% | 94.92% | 96.74% | 96.85% | 96.99% | 97.21% | 97.27% | 97.37% | 97.46% | 67.46% | 84.32% | 94.04% | 96.06% | 96.55% | 96.90% | 96.88% | 97.05% | 97.18% | 97.41% | **97.64%** |
|  | 0.55 | - | - | 64.67% | 77.61% | 92.72% | 94.95% | 96.67% | 96.87% | 96.96% | 96.97% | 97.12% | 97.09% | 97.07% | 67.26% | 82.12% | 93.29% | 95.85% | 96.50% | 96.60% | 96.75% | 96.90% | 97.13% | **97.35%** | 97.28% |
|  | 0.6 | - | - | 63.74% | 76.05% | 91.85% | 95.20% | 95.91% | 95.93% | 95.91% | 96.03% | 97.04% | **97.08%** | 97.04% | 66.64% | 82.86% | 93.19% | 95.02% | 95.64% | 95.80% | 95.89% | 96.15% | 96.41% | 96.67% | 96.84% |
|  | 0.65 | - | - | 62.55% | 75.37% | 89.86% | 93.25% | 93.81% | 94.06% | 94.16% | 94.08% | 94.13% | 94.20% | 94.25% | 65.55% | 81.04% | 91.15% | 93.68% | 94.61% | 95.03% | 95.14% | 95.27% | 95.35% | 95.31% | **95.49%** |
|  | 0.7 | - | - | 60.78% | 71.64% | 85.81% | 88.47% | 90.11% | 90.33% | 90.52% | 90.46% | 90.38% | 90.54% | 90.63% | 63.16% | 77.88% | 88.17% | 90.33% | 90.98% | 91.53% | 91.95% | 92.08% | 92.34% | 92.42% | **92.70%** |
|  | 0.75 | - | - | 55.90% | 66.07% | 78.20% | 81.57% | 81.87% | 82.03% | 82.19% | 82.15% | 82.28% | 82.30% | 82.34% | 58.71% | 72.65% | 81.83% | 84.38% | 85.07% | 85.32% | 85.40% | 85.63% | 85.76% | 85.83% | **86.04%** |
|  | 0.8 | - | - | 46.02% | 53.91% | 63.33% | 65.47% | 66.13% | 66.15% | 66.24% | 66.32% | 67.08% | 66.30% | 66.27% | 48.75% | 59.08% | 67.69% | 69.88% | 70.80% | 71.33% | 71.59% | 71.76% | 72.12% | 72.46% | **72.51%** |
|  | 0.85 | - | - | 29.81% | 34.52% | 39.38% | 40.92% | 41.48% | 41.75% | 41.85% | 41.76% | 41.75% | 42.36% | 41.79% | 31.11% | 39.90% | 45.81% | 47.99% | 48.95% | 49.22% | 49.60% | 49.93% | 50.19% | 50.31% | **50.45%** |
|  | 0.9 | - | - | 8.91% | 10.16% | 11.04% | 11.40% | 11.65% | 11.79% | 11.72% | 11.72% | 11.76% | 11.77% | 11.75% | 10.31% | 14.82% | 17.85% | 19.14% | 19.78% | 20.17% | 20.45% | 20.78% | 20.96% | 21.10% | **21.22%** |
|  | 0.95 | - | - | 0.25% | 0.33% | 0.30% | 0.32% | 0.35% | 0.34% | 0.34% | 0.35% | 0.34% | 0.34% | 0.34% | 0.38% | 2.02% | 2.58% | 3.05% | 3.23% | 3.51% | 3.76% | 3.61% | 3.55% | 3.71% | **3.71%** |
|  | 0.5:0.95 | 56.50% | 58.20% | 45.73% | 54.29% | 64.48% | 66.65% | 67.47% | 67.61% | 67.69% | 67.71% | 67.92% | 67.94% | 67.89% | 47.93% | 59.67% | 67.56% | 69.54% | 70.21% | 70.54% | 70.74% | 70.92% | 71.10% | 71.26% | **71.39%** |
| *C.albicans* | 0.5 | - | - | 87.93% | 89.14% | 90.14% | 90.19% | 90.26% | 91.13% | 91.21% | 91.19% | 91.22% | 91.19% | 91.20% | 87.54% | 91.82% | 93.24% | 94.19% | 95.13% | 95.41% | 95.64% | 95.85% | 95.99% | 96.18% | **96.26%** |
|  | 0.55 | - | - | 85.92% | 87.04% | 87.90% | 87.90% | 87.89% | 87.86% | 88.01% | 88.05% | 88.01% | 88.00% | 88.02% | 86.87% | 91.19% | 92.73% | 93.52% | 94.03% | 94.44% | 94.50% | 94.67% | 94.68% | 94.68% | **94.82%** |
|  | 0.6 | - | - | 83.60% | 83.66% | 83.61% | 84.48% | 84.38% | 84.46% | 84.52% | 84.50% | 84.44% | 84.47% | 84.47% | 85.37% | 89.17% | 90.48% | 91.17% | 91.76% | 92.05% | 92.16% | 92.23% | 92.36% | 92.47% | **92.61%** |
|  | 0.65 | - | - | 77.51% | 78.26% | 77.95% | 78.01% | 77.94% | 78.68% | 78.80% | 78.73% | 78.73% | 78.72% | 78.71% | 81.53% | 85.07% | 86.06% | 86.79% | 87.26% | 87.50% | 87.52% | 87.39% | 87.55% | **87.61%** | 87.59% |
|  | 0.7 | - | - | 70.21% | 70.00% | 69.71% | 70.39% | 70.33% | 70.11% | 70.03% | 70.08% | 70.05% | 69.98% | 69.96% | 73.60% | 76.07% | 77.57% | 77.54% | 78.29% | 78.63% | 78.90% | **79.18%** | 79.05% | 79.09% | 79.05% |
|  | 0.75 | - | - | 58.24% | 57.86% | 57.80% | 57.53% | 57.25% | 57.17% | 57.06% | 56.89% | 56.89% | 56.92% | 56.88% | 61.26% | 64.20% | 65.49% | 66.31% | 67.23% | 67.55% | 67.62% | **67.65%** | 67.63% | 67.65% | 67.64% |
|  | 0.8 | - | - | 40.82% | 40.38% | 39.76% | 39.42% | 38.88% | 39.34% | 38.57% | 39.06% | 38.52% | 38.50% | 38.46% | 42.14% | 44.36% | 46.54% | 48.08% | 48.97% | 49.32% | 49.48% | 49.56% | **49.86%** | 49.80% | 49.77% |
|  | 0.85 | - | - | 19.55% | 18.90% | 18.54% | 18.51% | 17.91% | 17.75% | 17.61% | 17.58% | 17.52% | 17.52% | 17.44% | 21.01% | 23.23% | 24.47% | 25.33% | 26.37% | 27.36% | 27.95% | 27.96% | 28.03% | **28.05%** | 27.91% |
|  | 0.9 | - | - | 3.39% | 3.28% | 3.18% | 3.12% | 3.12% | 3.12% | 3.13% | 3.11% | 3.15% | 3.11% | 3.06% | 4.11% | 5.99% | 6.95% | 7.62% | 7.81% | 8.17% | 8.46% | 8.61% | 8.65% | 8.72% | **8.78%** |
|  | 0.95 | - | - | 0.02% | 0.02% | 0.02% | 0.02% | 0.02% | 0.02% | 0.02% | 0.02% | 0.02% | 0.02% | 0.02% | 0.06% | 0.26% | 0.31% | 0.28% | 0.28% | 0.33% | **0.34%** | 0.32% | 0.31% | 0.34% | 0.34% |
|  | 0.5:0.95 | 65.20% | **66.80%** | 52.72% | 52.85% | 52.86% | 52.96% | 52.80% | 52.96% | 52.90% | 52.92% | 52.86% | 52.84% | 52.82% | 54.35% | 57.14% | 58.38% | 59.08% | 59.71% | 60.08% | 60.26% | 60.34% | 60.41% | 60.46% | 60.48% |

*Table S3. ICC: MAE and sMAPE on AGAR higher-resolution subset*

| Species | Conf | MAE | | | | | | | | | | | sMAPE | | | | | | | | | | |
| --- | --- | --- | --- | --- | --- | --- | --- | --- | --- | --- | --- | --- | --- | --- | --- | --- | --- | --- | --- | --- | --- | --- | --- |
|  |  | Iter 0 | Iter 1 | Iter 2 | Iter 3 | Iter 4 | Iter 5 | Iter 6 | Iter 7 | Iter 8 | Iter 9 | Iter 10 | Iter 0 | Iter 1 | Iter 2 | Iter 3 | Iter 4 | Iter 5 | Iter 6 | Iter 7 | Iter 8 | Iter 9 | Iter 10 |
| *S.aureus* | 0.10 | 11.25 | 12.38 | 12.75 | 12.50 | 11.75 | 11.50 | 11.25 | 11.38 | 11.00 | 11.88 | 11.63 | 16.89% | 18.93% | 19.36% | 18.84% | 18.28% | 18.25% | 18.00% | 18.12% | 17.71% | 18.84% | 18.56% |
|  | 0.11 | 10.13 | 10.38 | 11.13 | 11.13 | 11.38 | 10.88 | 10.75 | 10.88 | 10.50 | 10.50 | 10.88 | 15.84% | 16.79% | 17.84% | 17.76% | 18.09% | 17.42% | 17.39% | 17.81% | 17.04% | 17.18% | 17.55% |
|  | 0.12 | 9.38 | 10.00 | 11.00 | 10.50 | 10.25 | 10.25 | 10.00 | 10.50 | 9.75 | 9.75 | 9.75 | 15.17% | 15.24% | 16.46% | 15.77% | 15.48% | 15.31% | 15.06% | 15.85% | 14.66% | 14.70% | 14.70% |
|  | 0.13 | 8.50 | 8.63 | 9.38 | 9.50 | 9.50 | 9.38 | 9.25 | 9.38 | 9.63 | 9.13 | 9.00 | 14.13% | 13.11% | 14.00% | 14.28% | 14.32% | 14.27% | 14.03% | 13.90% | 14.04% | 13.71% | 13.45% |
|  | 0.14 | 7.38 | 8.38 | 8.75 | 8.63 | 8.88 | 8.63 | 8.88 | 9.13 | 9.13 | 8.25 | 7.75 | 12.85% | 12.49% | 13.14% | 12.88% | 13.23% | 12.70% | 13.14% | 13.62% | 13.66% | 12.53% | 12.05% |
|  | 0.15 | 6.88 | 7.13 | 8.00 | 8.00 | 8.25 | 8.38 | 9.00 | 8.50 | 8.38 | 8.38 | 7.88 | 12.46% | 11.34% | 12.33% | 12.22% | 12.69% | 12.56% | 13.40% | 12.84% | 12.70% | 12.51% | 11.83% |
|  | 0.16 | 6.38 | 6.63 | 7.13 | 7.13 | 7.38 | 7.38 | 7.75 | 7.50 | 7.00 | 6.75 | 6.75 | 11.86% | 10.77% | 11.40% | 11.39% | 11.69% | 11.62% | 12.33% | 12.11% | 11.33% | 10.86% | 10.93% |
|  | 0.17 | 5.25 | 5.63 | 6.25 | 6.63 | 6.88 | 7.13 | 7.25 | 7.38 | 7.63 | 7.63 | 7.75 | 10.81% | 9.97% | 10.59% | 11.08% | 11.33% | 11.70% | 11.67% | 11.72% | 11.98% | 12.23% | 12.32% |
|  | 0.18 | 4.50 | 6.38 | 6.63 | 6.75 | 7.00 | 6.63 | 6.50 | 6.88 | 7.25 | 6.88 | 6.63 | 8.98% | 10.58% | 10.84% | 11.25% | 11.43% | 11.00% | 10.83% | 11.27% | 11.71% | 10.89% | 10.62% |
|  | 0.19 | 3.88 | 5.13 | 6.00 | 5.75 | 6.38 | 6.00 | 5.88 | 6.13 | 6.38 | 6.00 | 5.88 | 6.85% | 7.80% | 9.04% | 8.58% | 9.20% | 8.77% | 8.69% | 8.99% | 9.30% | 8.62% | 8.48% |
|  | 0.20 | 2.63 | 3.50 | 3.88 | 4.38 | 4.50 | 4.88 | 4.38 | 4.63 | 5.13 | 4.88 | 4.63 | 5.49% | 6.33% | 6.68% | 7.16% | 7.29% | 7.48% | 6.63% | 7.05% | 7.80% | 7.57% | 7.00% |
|  | 0.21 | 1.88 | 2.38 | 4.00 | 3.75 | 3.75 | 4.25 | 4.13 | 4.25 | 4.00 | 3.88 | 3.88 | 5.22% | 4.83% | 6.71% | 6.35% | 6.40% | 6.98% | 6.84% | 7.07% | 6.50% | 6.39% | 6.48% |
|  | 0.22 | **1.50** | 2.13 | 2.88 | 2.88 | 3.13 | 3.38 | 3.88 | 3.50 | 4.25 | 4.38 | 4.50 | **4.91%** | 4.83% | 5.49% | 5.49% | 5.74% | 5.84% | 6.41% | 5.68% | 6.93% | 7.11% | 7.02% |
|  | 0.23 | 1.50 | 1.63 | 2.25 | 2.75 | 3.00 | 3.00 | 3.25 | 3.25 | 3.00 | 3.38 | 3.50 | 5.52% | 3.96% | 4.89% | 5.30% | 5.72% | 5.73% | 5.97% | 5.83% | 5.25% | 5.83% | 5.88% |
|  | 0.24 | 1.50 | 1.25 | 1.63 | 1.88 | 2.25 | 2.38 | 2.75 | 2.25 | 2.50 | 2.75 | 3.13 | 5.29% | 3.65% | 4.15% | 4.31% | 4.66% | 4.76% | 5.06% | 4.37% | 4.85% | 5.01% | 5.60% |
|  | 0.25 | 1.75 | 0.63 | 1.13 | 1.38 | 1.63 | 1.50 | 1.63 | 1.75 | 1.38 | 1.75 | 2.13 | 5.39% | 2.60% | 3.59% | 3.74% | 4.10% | 4.08% | 4.19% | 4.25% | 3.47% | 4.02% | 4.47% |
|  | 0.26 | 2.25 | 1.25 | 0.88 | 0.88 | 0.38 | 0.63 | 0.63 | 0.50 | 0.88 | 0.88 | 1.13 | 5.66% | 3.30% | 2.96% | 2.96% | 2.43% | 2.79% | 2.80% | 2.49% | 3.15% | 3.00% | 3.36% |
|  | 0.27 | 2.63 | 1.88 | 1.75 | 1.38 | 1.50 | 1.25 | 1.38 | 1.38 | 0.88 | **0.38** | 0.63 | 6.15% | 3.63% | 4.09% | 3.63% | 3.83% | 3.79% | 3.95% | 3.87% | 3.15% | **2.34%** | 2.75% |
|  | 0.28 | 3.13 | 2.63 | 2.50 | 2.13 | 2.13 | 1.75 | 1.50 | 1.13 | 0.88 | 0.75 | 0.75 | 6.72% | 5.14% | 5.25% | 4.85% | 4.85% | 4.46% | 4.19% | 3.77% | 3.55% | 3.34% | 3.25% |
|  | 0.29 | 3.88 | 3.25 | 2.88 | 2.50 | 2.13 | 2.25 | 2.25 | 2.13 | 1.50 | 1.63 | 1.50 | 7.92% | 5.98% | 5.52% | 5.16% | 4.86% | 5.11% | 5.31% | 5.02% | 4.19% | 4.40% | 4.19% |
|  | 0.30 | 4.63 | 3.13 | 2.63 | 2.50 | 2.25 | 2.38 | 2.13 | 2.38 | 1.88 | 1.75 | 1.63 | 9.06% | 5.76% | 5.33% | 5.12% | 4.94% | 5.30% | 5.11% | 5.47% | 4.78% | 4.53% | 4.36% |
| *B.subtilis* | 0.10 | 1.38 | 0.88 | 1.25 | 1.25 | 1.25 | 1.25 | 1.00 | 1.13 | 0.88 | 1.00 | 1.25 | 6.53% | 0.91% | 2.27% | 2.27% | 2.27% | 2.27% | 2.02% | 2.14% | 1.89% | 2.02% | 2.27% |
|  | 0.11 | 1.25 | 0.88 | 1.13 | 1.00 | 1.00 | 1.25 | 1.25 | 1.25 | 1.13 | 1.88 | 1.75 | 6.82% | 1.27% | 1.83% | 1.71% | 1.71% | 1.96% | 1.96% | 1.96% | 1.83% | 2.56% | 2.44% |
|  | 0.12 | 1.25 | 0.75 | 0.88 | 1.00 | 0.88 | 0.75 | 0.75 | 0.50 | 0.50 | 0.50 | 0.50 | 6.82% | 1.14% | 1.25% | 1.38% | 1.25% | 1.13% | 1.13% | 0.87% | 0.87% | 0.87% | 0.87% |
|  | 0.13 | 1.25 | 1.00 | 0.63 | 0.63 | 0.63 | 0.63 | 0.38 | 0.38 | 0.88 | 0.63 | 0.63 | 6.82% | 2.19% | 0.65% | 0.65% | 0.65% | 0.65% | 0.40% | 0.40% | 0.91% | 0.65% | 0.65% |
|  | 0.14 | 1.25 | 0.88 | 0.75 | 0.75 | 0.75 | 0.75 | 0.50 | 0.50 | 0.50 | 0.50 | 0.38 | 6.82% | 2.06% | 1.14% | 1.14% | 1.14% | 1.14% | 0.89% | 0.89% | 0.89% | 0.89% | 0.76% |
|  | 0.15 | 1.25 | 0.88 | 0.75 | 0.75 | 0.63 | 0.63 | 0.63 | 0.38 | 0.25 | 0.38 | 0.25 | 6.82% | 2.06% | 1.14% | 1.14% | 1.02% | 1.02% | 1.02% | 0.76% | 0.62% | 0.76% | 0.62% |
|  | 0.16 | 1.25 | 0.75 | 0.50 | 0.75 | 0.75 | 0.63 | 0.50 | 0.50 | 0.50 | 0.50 | 0.38 | 6.82% | 1.93% | 0.89% | 1.14% | 1.14% | 1.02% | 0.89% | 0.89% | 0.89% | 0.89% | 0.76% |
|  | 0.17 | 1.00 | 0.75 | 0.38 | 0.50 | 0.38 | 0.38 | 0.38 | 0.38 | 0.38 | 0.38 | 0.50 | 6.56% | 1.93% | 0.76% | 0.89% | 0.76% | 0.76% | 0.76% | 0.76% | 0.76% | 0.76% | 0.89% |
|  | 0.18 | 0.88 | 0.63 | 0.50 | 0.50 | 0.63 | 0.50 | 0.25 | 0.25 | 0.25 | 0.25 | 0.25 | 6.42% | 1.80% | 0.89% | 0.89% | 1.02% | 0.89% | 0.62% | 0.62% | 0.62% | 0.62% | 0.62% |
|  | 0.19 | 0.88 | 0.38 | 0.38 | 0.38 | 0.38 | 0.25 | 0.25 | 0.25 | 0.25 | 0.25 | 0.25 | 3.78% | 1.53% | 0.76% | 0.76% | 0.76% | 0.62% | 0.62% | 0.62% | 0.62% | 0.62% | 0.62% |
|  | 0.20 | **0.75** | 0.38 | **0.13** | 0.13 | 0.13 | 0.38 | 0.25 | 0.75 | 0.75 | 0.63 | 0.63 | **3.02%** | 1.53% | **0.49%** | 0.49% | 0.49% | 0.76% | 0.62% | 1.14% | 1.14% | 1.02% | 1.02% |
|  | 0.21 | 0.88 | 0.50 | 0.25 | 0.25 | 0.25 | 0.13 | 0.25 | 0.25 | 0.25 | 0.25 | 0.25 | 3.16% | 1.67% | 0.63% | 0.63% | 0.63% | 0.49% | 0.63% | 0.63% | 0.63% | 0.63% | 0.63% |
|  | 0.22 | 0.88 | 0.50 | 0.25 | 0.25 | 0.25 | 0.25 | 0.25 | 0.25 | 0.25 | 0.25 | 0.25 | 3.16% | 1.67% | 0.63% | 0.63% | 0.63% | 0.63% | 0.63% | 0.63% | 0.63% | 0.63% | 0.63% |
|  | 0.23 | 0.88 | 0.50 | 0.25 | 0.25 | 0.25 | 0.25 | 0.25 | 0.25 | 0.25 | 0.25 | 0.25 | 3.16% | 1.67% | 0.63% | 0.63% | 0.63% | 0.63% | 0.63% | 0.63% | 0.63% | 0.63% | 0.63% |
|  | 0.24 | 0.88 | 0.50 | 0.25 | 0.25 | 0.38 | 0.38 | 0.38 | 0.38 | 0.38 | 0.38 | 0.38 | 3.16% | 1.67% | 0.63% | 0.63% | 0.76% | 0.76% | 0.76% | 0.76% | 0.76% | 0.76% | 0.76% |
|  | 0.25 | 1.13 | 0.75 | 0.50 | 0.50 | 0.38 | 0.38 | 0.38 | 0.38 | 0.38 | 0.38 | 0.38 | 3.44% | 2.36% | 0.90% | 0.90% | 0.76% | 0.76% | 0.76% | 0.76% | 0.76% | 0.76% | 0.76% |
|  | 0.26 | 1.13 | 0.88 | 0.50 | 0.50 | 0.38 | 0.38 | 0.38 | 0.38 | 0.38 | 0.38 | 0.38 | 2.77% | 2.49% | 0.90% | 0.90% | 0.76% | 0.76% | 0.76% | 0.76% | 0.76% | 0.76% | 0.76% |
|  | 0.27 | 1.13 | 1.00 | 0.38 | 0.38 | 0.38 | 0.38 | 0.38 | 0.38 | 0.38 | 0.38 | 0.38 | 2.77% | 2.63% | 0.76% | 0.76% | 0.76% | 0.76% | 0.76% | 0.76% | 0.76% | 0.76% | 0.76% |
|  | 0.28 | 1.38 | 1.13 | 0.63 | 0.63 | 0.63 | 0.50 | 0.50 | 0.50 | 0.50 | 0.50 | 0.50 | 3.49% | 2.77% | 1.41% | 1.41% | 1.41% | 1.27% | 1.27% | 1.27% | 1.27% | 1.27% | 1.27% |
|  | 0.29 | 1.50 | 1.13 | 0.88 | 0.63 | 0.63 | 0.63 | 0.63 | 0.63 | 0.50 | 0.50 | 0.50 | 3.64% | 2.77% | 1.69% | 1.41% | 1.41% | 1.41% | 1.41% | 1.41% | 1.27% | 1.27% | 1.27% |
|  | 0.30 | 1.50 | 1.38 | 0.88 | 0.88 | 0.88 | 0.88 | 0.88 | 0.88 | 0.88 | 0.75 | 0.75 | 3.64% | 3.06% | 2.08% | 2.08% | 2.08% | 2.08% | 2.08% | 2.08% | 2.08% | 1.94% | 1.94% |
| *P.aeruginosa* | 0.10 | 8.63 | 9.88 | 12.75 | 12.63 | 12.63 | 13.25 | 13.63 | 15.38 | 16.13 | 16.75 | 17.75 | 8.39% | 10.68% | 8.63% | 8.05% | 7.63% | 8.58% | 8.05% | 9.81% | 10.64% | 11.09% | 11.73% |
|  | 0.11 | 6.63 | 5.75 | 4.50 | 7.13 | 9.00 | 11.63 | 12.38 | 12.88 | 14.13 | 14.75 | 15.38 | 7.75% | 3.88% | 5.63% | 4.02% | 5.22% | 7.06% | 6.94% | 8.15% | 9.19% | 9.70% | 10.11% |
|  | 0.12 | 5.63 | 4.88 | 3.75 | 6.38 | 8.00 | 8.50 | 9.88 | 11.00 | 12.50 | 12.75 | 14.50 | 7.79% | 4.02% | 3.01% | 4.68% | 6.61% | 4.87% | 6.15% | 6.12% | 8.23% | 8.47% | 10.10% |
|  | 0.13 | 5.00 | 4.50 | 7.00 | 6.38 | 6.88 | 8.25 | 8.75 | 9.25 | 10.25 | 11.38 | 12.63 | 8.55% | 4.64% | 7.81% | 5.42% | 5.01% | 8.77% | 8.03% | 6.32% | 6.45% | 7.54% | 8.37% |
|  | 0.14 | **4.25** | 4.00 | 3.75 | 3.88 | 6.13 | 6.00 | 8.38 | 9.00 | 10.50 | 11.88 | 13.25 | **8.30%** | 5.51% | 3.67% | 3.33% | 5.86% | 3.84% | 5.34% | 5.49% | 6.74% | 7.58% | 9.01% |
|  | 0.15 | 5.38 | 3.63 | 5.38 | 5.88 | 6.63 | 7.38 | 6.75 | 7.50 | 9.50 | 10.13 | 10.88 | 9.10% | 5.74% | 8.22% | 5.89% | 4.49% | 6.80% | 4.80% | 4.49% | 6.00% | 6.65% | 7.22% |
|  | 0.16 | 6.25 | 3.88 | 4.00 | 3.00 | 3.00 | 3.88 | 3.75 | 4.75 | 6.63 | 8.25 | 8.38 | 10.78% | 5.82% | 6.55% | 3.70% | 2.51% | 4.66% | 2.50% | 3.18% | 4.74% | 5.69% | 5.50% |
|  | 0.17 | 6.38 | 4.38 | 3.38 | 4.75 | 5.63 | 6.75 | 5.25 | 5.25 | 5.75 | 6.38 | 6.88 | 10.87% | 7.95% | 4.27% | 5.95% | 6.02% | 10.51% | 7.12% | 5.49% | 4.63% | 4.07% | 4.41% |
|  | 0.18 | 7.25 | 4.88 | 5.50 | 4.75 | 4.38 | 3.63 | 4.25 | 3.50 | 3.38 | 4.63 | 5.00 | 11.62% | 6.56% | 8.79% | 6.80% | 4.57% | 4.55% | 3.92% | 2.22% | 2.47% | 3.39% | 3.68% |
|  | 0.19 | 9.25 | 6.63 | 4.88 | 3.50 | 3.75 | 4.25 | 3.50 | 3.63 | 3.38 | 3.75 | 4.75 | 13.75% | 9.11% | 5.18% | 5.40% | 7.52% | 5.05% | 2.84% | 3.20% | 1.85% | 2.16% | 3.56% |
|  | 0.20 | 10.50 | 7.50 | 8.75 | 6.50 | 6.38 | 4.25 | 3.88 | 3.25 | 2.38 | 2.38 | 2.00 | 14.60% | 11.93% | 15.70% | 8.51% | 7.67% | 4.91% | 3.70% | 4.59% | 2.42% | 2.12% | 1.75% |
|  | 0.21 | 11.63 | 8.88 | 7.25 | 6.00 | 7.25 | 5.50 | 4.25 | 4.13 | 1.75 | **1.25** | 1.75 | 15.98% | 10.79% | 8.30% | 8.06% | 10.90% | 6.62% | 4.32% | 4.16% | 1.73% | **1.01%** | 1.73% |
|  | 0.22 | 12.50 | 9.63 | 9.38 | 8.75 | 7.50 | 7.75 | 5.13 | 2.63 | 2.50 | 2.25 | 2.38 | 16.63% | 11.48% | 13.05% | 8.26% | 5.81% | 9.78% | 5.99% | 2.66% | 1.99% | 2.14% | 1.90% |
|  | 0.23 | 13.00 | 12.38 | 12.63 | 10.63 | 8.88 | 8.75 | 7.00 | 4.25 | 3.75 | 2.38 | 1.63 | 17.13% | 14.72% | 18.85% | 12.33% | 9.52% | 14.48% | 9.63% | 4.30% | 4.09% | 2.25% | 1.37% |
|  | 0.24 | 13.88 | 12.00 | 9.75 | 7.88 | 7.00 | 6.50 | 5.25 | 5.38 | 3.50 | 3.13 | 2.63 | 18.94% | 21.03% | 14.91% | 10.79% | 10.95% | 9.17% | 6.39% | 7.24% | 4.36% | 3.72% | 3.20% |
|  | 0.25 | 15.13 | 12.75 | 10.88 | 9.88 | 8.88 | 8.25 | 8.50 | 7.25 | 5.25 | 5.50 | 5.00 | 20.61% | 15.95% | 14.92% | 13.86% | 10.90% | 9.47% | 11.84% | 8.95% | 6.13% | 5.85% | 5.49% |
|  | 0.26 | 16.13 | 14.00 | 13.00 | 12.38 | 12.88 | 12.50 | 10.00 | 9.38 | 7.13 | 6.50 | 6.00 | 22.04% | 18.44% | 14.63% | 15.42% | 15.65% | 19.87% | 16.07% | 13.87% | 8.51% | 7.67% | 6.15% |
|  | 0.27 | 17.63 | 16.88 | 16.63 | 13.50 | 12.63 | 11.75 | 10.50 | 9.25 | 7.63 | 6.88 | 5.13 | 24.08% | 22.39% | 23.77% | 16.41% | 15.30% | 17.63% | 13.66% | 10.40% | 8.83% | 7.82% | 5.34% |
|  | 0.28 | 18.88 | 17.00 | 16.13 | 14.75 | 14.13 | 13.13 | 11.13 | 10.50 | 8.50 | 7.63 | 7.13 | 25.57% | 20.67% | 19.35% | 19.33% | 18.75% | 21.85% | 18.22% | 15.47% | 12.59% | 10.09% | 8.69% |
|  | 0.29 | 20.38 | 20.63 | 18.13 | 18.38 | 16.75 | 13.75 | 13.13 | 13.13 | 10.75 | 10.00 | 9.13 | 29.12% | 27.80% | 26.65% | 28.70% | 26.14% | 18.09% | 16.25% | 18.98% | 14.70% | 13.64% | 12.00% |
|  | 0.30 | 22.38 | 18.63 | 18.63 | 17.50 | 16.75 | 16.75 | 14.25 | 13.00 | 11.88 | 11.00 | 10.38 | 32.14% | 26.64% | 29.19% | 27.84% | 25.37% | 28.19% | 22.72% | 20.02% | 16.13% | 14.64% | 13.27% |
| *E.coli* | 0.10 | 13.88 | 16.25 | 19.38 | 21.63 | 22.50 | 22.75 | 23.38 | 24.25 | 24.13 | 24.88 | 25.00 | 15.26% | 15.88% | 18.11% | 19.41% | 19.79% | 19.92% | 20.36% | 20.92% | 20.93% | 21.36% | 21.38% |
|  | 0.11 | 10.38 | 11.88 | 14.63 | 17.13 | 17.00 | 18.75 | 19.63 | 20.25 | 21.00 | 21.63 | 21.63 | 13.23% | 13.09% | 14.82% | 16.34% | 16.09% | 17.46% | 17.91% | 18.49% | 18.90% | 19.46% | 19.46% |
|  | 0.12 | 7.38 | 9.13 | 10.38 | 13.38 | 14.38 | 15.63 | 16.75 | 17.50 | 17.38 | 18.00 | 18.50 | 8.08% | 8.11% | 8.79% | 10.69% | 11.51% | 12.46% | 13.28% | 13.78% | 13.61% | 14.08% | 14.30% |
|  | 0.13 | 6.00 | 6.63 | 6.00 | 8.25 | 10.88 | 12.38 | 13.38 | 14.38 | 15.00 | 15.88 | 16.00 | 5.19% | 4.27% | 3.61% | 5.27% | 6.99% | 8.04% | 8.78% | 9.58% | 10.04% | 10.62% | 10.68% |
|  | 0.14 | 5.25 | 6.63 | 8.38 | 9.00 | 11.50 | 12.38 | 13.13 | 14.38 | 14.75 | 14.88 | 15.63 | 4.73% | 4.44% | 5.96% | 6.23% | 7.81% | 8.50% | 9.03% | 9.88% | 10.12% | 10.10% | 10.56% |
|  | 0.15 | 5.13 | 6.00 | 8.00 | 8.50 | 10.25 | 10.13 | 10.88 | 12.00 | 12.13 | 12.38 | 13.13 | 4.80% | 4.10% | 5.47% | 5.42% | 6.73% | 6.79% | 7.31% | 8.18% | 8.31% | 8.54% | 9.03% |
|  | 0.16 | 4.63 | 3.38 | 4.50 | 7.25 | 8.50 | 9.50 | 10.75 | 11.38 | 12.00 | 12.50 | 12.88 | 4.63% | 2.38% | 3.21% | 5.04% | 5.90% | 6.55% | 7.37% | 7.78% | 8.21% | 8.52% | 8.74% |
|  | 0.17 | 5.25 | 3.75 | 4.25 | 6.75 | 6.38 | 7.38 | 8.25 | 9.75 | 11.00 | 11.38 | 11.38 | 7.11% | 4.58% | 4.74% | 6.42% | 6.02% | 6.81% | 7.58% | 8.67% | 9.53% | 9.74% | 9.68% |
|  | 0.18 | 5.25 | 4.75 | 5.00 | 4.50 | 5.13 | 5.25 | 6.38 | 7.63 | 8.75 | 9.13 | 10.00 | 7.37% | 5.41% | 5.51% | 4.96% | 5.26% | 5.23% | 6.14% | 7.11% | 7.87% | 8.06% | 8.67% |
|  | 0.19 | 5.50 | 4.50 | 4.88 | 4.13 | 4.25 | 4.50 | 4.88 | 5.50 | 6.50 | 7.63 | 8.13 | 7.77% | 5.50% | 5.71% | 4.82% | 4.75% | 4.82% | 5.11% | 5.66% | 6.36% | 7.23% | 7.59% |
|  | 0.20 | 5.25 | 4.13 | 4.75 | 3.00 | 3.50 | 4.13 | 4.75 | 4.88 | 5.88 | 6.38 | 6.50 | 7.75% | 5.30% | 5.78% | 3.86% | 4.20% | 4.75% | 5.28% | 5.42% | 6.05% | 6.44% | 6.44% |
|  | 0.21 | **4.38** | 4.50 | 5.13 | 4.13 | 3.63 | 2.75 | 2.88 | 3.00 | 4.25 | 5.38 | 5.00 | **7.29%** | 5.82% | 6.18% | 5.05% | 4.38% | 3.58% | 3.68% | 3.75% | 4.70% | 5.61% | 5.23% |
|  | 0.22 | 5.38 | 5.75 | 4.25 | 3.50 | 2.88 | 2.63 | 2.38 | 2.63 | 3.00 | 3.00 | 3.00 | 6.90% | 7.30% | 6.14% | 5.21% | 4.37% | 3.89% | 3.57% | 3.66% | 3.92% | 3.85% | 3.85% |
|  | 0.23 | 6.38 | 4.13 | 3.38 | 3.75 | 3.38 | 2.88 | 2.38 | 2.88 | 3.00 | 3.25 | 3.00 | 8.06% | 5.55% | 4.87% | 4.97% | 4.44% | 3.93% | 3.49% | 3.81% | 3.80% | 4.03% | 3.80% |
|  | 0.24 | 7.38 | 6.13 | 5.25 | 3.75 | 3.25 | 3.38 | 2.25 | 1.88 | 1.88 | 1.75 | 2.25 | 10.44% | 7.62% | 6.96% | 5.49% | 4.76% | 4.74% | 3.82% | 3.44% | 3.43% | 3.22% | 3.64% |
|  | 0.25 | 8.25 | 7.25 | 6.63 | 5.13 | 4.75 | 3.63 | 2.50 | 1.75 | 1.25 | **1.00** | 1.50 | 11.35% | 10.12% | 9.74% | 8.20% | 7.89% | 6.66% | 5.51% | 4.78% | 4.25% | **4.04%** | 4.47% |
|  | 0.26 | 9.00 | 7.75 | 7.38 | 5.50 | 4.25 | 3.63 | 3.25 | 3.25 | 2.50 | 2.00 | 2.13 | 14.23% | 12.59% | 12.30% | 10.38% | 9.24% | 8.51% | 8.28% | 8.28% | 7.61% | 7.21% | 7.38% |
|  | 0.27 | 9.63 | 8.50 | 8.50 | 7.25 | 5.75 | 5.25 | 4.63 | 4.00 | 3.50 | 3.63 | 3.38 | 16.36% | 15.19% | 15.31% | 13.84% | 12.38% | 11.77% | 11.17% | 10.53% | 10.06% | 10.13% | 9.95% |
|  | 0.28 | 10.25 | 9.25 | 7.38 | 6.00 | 5.50 | 5.63 | 5.25 | 4.75 | 4.38 | 4.25 | 3.88 | 17.02% | 16.05% | 13.90% | 12.53% | 12.00% | 12.15% | 11.82% | 11.27% | 10.86% | 10.74% | 10.48% |
|  | 0.29 | 10.88 | 10.38 | 9.13 | 8.13 | 6.88 | 6.38 | 5.75 | 5.13 | 4.88 | 4.88 | 4.63 | 17.70% | 16.99% | 15.59% | 14.84% | 13.43% | 12.91% | 12.21% | 11.59% | 11.39% | 11.39% | 11.15% |
|  | 0.30 | 12.25 | 11.00 | 10.38 | 8.13 | 7.13 | 6.38 | 5.63 | 5.38 | 5.00 | 5.00 | 5.00 | 19.34% | 17.89% | 17.33% | 14.88% | 13.73% | 12.92% | 12.15% | 11.84% | 11.45% | 11.45% | 11.45% |
| *C.albicans* | 0.10 | 6.88 | 5.38 | 6.38 | 6.50 | 6.25 | 6.38 | 6.88 | 6.75 | 6.63 | 6.50 | 6.63 | 28.40% | 5.97% | 8.51% | 9.80% | 9.94% | 9.76% | 10.06% | 9.98% | 9.91% | 9.60% | 9.67% |
|  | 0.11 | 6.63 | 5.25 | 6.38 | 6.13 | 6.00 | 5.75 | 5.88 | 5.88 | 5.88 | 6.25 | 6.38 | 28.54% | 7.58% | 7.43% | 7.95% | 8.55% | 7.62% | 7.46% | 7.46% | 7.46% | 8.17% | 8.25% |
|  | 0.12 | 6.50 | 5.38 | 5.75 | 5.88 | 5.38 | 5.13 | 5.25 | 5.38 | 5.50 | 6.00 | 5.75 | 28.76% | 8.27% | 7.90% | 7.79% | 7.89% | 7.00% | 7.07% | 7.15% | 7.47% | 8.02% | 7.87% |
|  | 0.13 | 6.25 | 5.38 | 4.88 | 4.63 | 4.75 | 4.63 | 5.13 | 5.13 | 5.50 | 5.63 | 5.50 | 32.73% | 13.18% | 6.34% | 4.42% | 6.82% | 6.74% | 7.06% | 7.06% | 7.53% | 7.86% | 7.78% |
|  | 0.14 | 6.13 | 5.63 | 5.13 | 5.00 | 4.38 | 4.63 | 4.50 | 4.50 | 4.50 | 5.00 | 4.75 | 32.37% | 11.78% | 5.80% | 6.36% | 5.42% | 4.85% | 4.77% | 4.77% | 5.01% | 5.57% | 5.41% |
|  | 0.15 | 5.63 | 3.38 | 4.00 | 3.63 | 3.50 | 3.00 | 3.13 | 2.88 | 3.13 | 3.50 | 3.63 | 32.16% | 8.76% | 6.42% | 4.19% | 4.11% | 3.05% | 3.13% | 2.96% | 3.60% | 4.11% | 4.19% |
|  | 0.16 | 5.13 | 3.38 | 3.13 | 2.88 | 3.13 | 2.75 | 2.75 | 2.75 | 3.00 | 3.63 | 3.88 | 31.80% | 8.49% | 3.91% | 2.90% | 3.85% | 3.34% | 3.34% | 3.34% | 3.51% | 4.19% | 4.36% |
|  | 0.17 | 4.75 | 4.00 | 3.25 | 2.25 | 2.63 | 2.38 | 2.50 | 2.75 | 2.75 | 3.00 | 2.88 | 34.76% | 14.34% | 8.15% | 2.44% | 2.72% | 1.83% | 1.92% | 2.10% | 2.56% | 2.99% | 2.90% |
|  | 0.18 | 4.50 | 2.63 | 2.50 | 2.00 | 1.75 | 2.00 | 1.88 | 2.38 | 2.25 | 2.50 | 2.88 | 35.22% | 8.20% | 6.67% | 4.24% | 2.90% | 2.85% | 2.76% | 3.13% | 3.04% | 3.46% | 3.73% |
|  | 0.19 | 4.38 | 2.38 | 2.25 | 1.63 | 1.88 | 1.88 | 2.00 | 2.38 | 2.50 | 2.75 | 3.00 | 34.67% | 8.27% | 6.03% | 3.66% | 4.50% | 4.02% | 4.12% | 4.40% | 4.49% | 5.17% | 5.35% |
|  | 0.20 | 4.38 | 2.25 | 2.00 | 2.00 | 1.50 | 1.50 | 1.75 | 1.75 | 2.13 | 2.88 | 3.13 | 35.05% | 12.26% | 10.63% | 9.56% | 7.69% | 7.46% | 7.66% | 7.66% | 7.95% | 9.00% | 9.18% |
|  | 0.21 | **4.25** | 3.75 | 3.13 | 2.25 | 1.88 | 1.75 | 1.75 | 1.50 | 1.75 | 2.00 | 2.00 | **38.81%** | 17.08% | 14.63% | 9.60% | 7.59% | 6.99% | 6.99% | 6.80% | 7.24% | 7.43% | 7.43% |
|  | 0.22 | 4.63 | 2.25 | 2.00 | 1.75 | 1.63 | 1.50 | 1.75 | 1.63 | 1.88 | 1.88 | 2.13 | 39.53% | 14.38% | 10.63% | 9.36% | 7.39% | 6.80% | 6.99% | 6.89% | 7.59% | 7.33% | 7.53% |
|  | 0.23 | 5.13 | 4.25 | 2.25 | 1.75 | 1.63 | 1.00 | 1.25 | 1.38 | 1.75 | 1.75 | 1.75 | 41.28% | 28.90% | 14.09% | 9.19% | 8.81% | 7.29% | 7.49% | 7.59% | 8.39% | 8.39% | 8.39% |
|  | 0.24 | 5.50 | 1.75 | 1.50 | 1.63 | 1.63 | 1.75 | 1.75 | 1.75 | 1.75 | 1.88 | 1.63 | 41.60% | 11.60% | 10.22% | 9.26% | 8.29% | 8.39% | 8.39% | 8.39% | 8.39% | 8.49% | 8.29% |
|  | 0.25 | 6.50 | 2.75 | 1.88 | 1.38 | **1.00** | 1.00 | 1.13 | 1.00 | 1.25 | 1.25 | 1.25 | 44.51% | 17.49% | 13.23% | 9.31% | **7.29%** | 7.29% | 7.39% | 7.29% | 7.98% | 7.98% | 7.98% |
|  | 0.26 | 7.38 | 5.75 | 2.13 | 2.00 | 2.00 | 1.13 | 1.25 | 1.25 | 1.25 | 1.38 | 1.38 | 47.10% | 25.68% | 12.20% | 11.03% | 10.06% | 8.04% | 8.15% | 8.15% | 8.63% | 8.74% | 8.74% |
|  | 0.27 | 8.13 | 2.63 | 2.50 | 2.38 | 1.63 | 1.88 | 1.75 | 1.88 | 1.88 | 1.63 | 1.63 | 49.06% | 15.06% | 12.25% | 11.08% | 8.70% | 9.16% | 9.06% | 9.16% | 9.16% | 8.95% | 8.95% |
|  | 0.28 | 8.75 | 4.00 | 3.00 | 2.63 | 2.50 | 2.38 | 2.38 | 2.38 | 2.38 | 1.88 | 1.75 | 50.34% | 17.44% | 13.85% | 11.57% | 10.49% | 10.11% | 10.11% | 10.11% | 10.11% | 9.16% | 9.06% |
|  | 0.29 | 9.25 | 6.38 | 4.00 | 3.25 | 2.75 | 2.63 | 2.38 | 2.38 | 2.25 | 2.13 | 2.13 | 51.54% | 34.41% | 20.98% | 14.34% | 13.12% | 13.01% | 12.78% | 12.78% | 12.93% | 12.57% | 12.57% |
|  | 0.30 | 10.00 | 4.38 | 3.00 | 2.88 | 2.75 | 2.63 | 2.63 | 2.50 | 2.38 | 2.00 | 1.88 | 54.60% | 19.61% | 13.59% | 12.32% | 11.16% | 10.08% | 10.08% | 9.97% | 9.85% | 9.27% | 9.16% |

*Table S4. ICC: MAE and sMAPE on AGAR lower-resolution subset*

| Species | Conf | MAE | | | | | | | | | | | sMAPE | | | | | | | | | | |
| --- | --- | --- | --- | --- | --- | --- | --- | --- | --- | --- | --- | --- | --- | --- | --- | --- | --- | --- | --- | --- | --- | --- | --- |
|  |  | Iter 0 | Iter 1 | Iter 2 | Iter 3 | Iter 4 | Iter 5 | Iter 6 | Iter 7 | Iter 8 | Iter 9 | Iter 10 | Iter 0 | Iter 1 | Iter 2 | Iter 3 | Iter 4 | Iter 5 | Iter 6 | Iter 7 | Iter 8 | Iter 9 | Iter 10 |
| *S.aureus* | 0.10 | 0.75 | 1.00 | 0.88 | 0.88 | 1.38 | 1.13 | 1.13 | 1.00 | 1.00 | 1.00 | 1.00 | 1.61% | 1.99% | 1.80% | 1.80% | 2.53% | 2.17% | 2.17% | 1.99% | 1.99% | 1.99% | 1.99% |
|  | 0.11 | 0.75 | 0.88 | 1.25 | 1.00 | 1.00 | 1.00 | 1.00 | 1.00 | 1.00 | 1.00 | 1.00 | 1.61% | 1.80% | 2.35% | 1.99% | 1.99% | 1.99% | 1.99% | 1.99% | 1.99% | 1.99% | 1.99% |
|  | 0.12 | 0.75 | 0.88 | 1.00 | 1.00 | 1.00 | 1.00 | 1.00 | 0.88 | 0.88 | 0.88 | 1.00 | 1.61% | 1.80% | 1.99% | 1.99% | 1.99% | 1.99% | 1.99% | 1.80% | 1.80% | 1.80% | 1.99% |
|  | 0.13 | 0.63 | 0.50 | 0.75 | 0.75 | 0.75 | 0.75 | 0.75 | 0.75 | 0.75 | 0.75 | 1.00 | 1.35% | 1.16% | 1.61% | 1.61% | 1.61% | 1.61% | 1.61% | 1.61% | 1.61% | 1.61% | 1.99% |
|  | 0.14 | 0.63 | 0.75 | 1.00 | 1.00 | 0.88 | 0.88 | 0.88 | 0.88 | 0.88 | 0.88 | 0.88 | 1.35% | 1.54% | 1.91% | 1.99% | 1.72% | 1.72% | 1.72% | 1.72% | 1.72% | 1.72% | 1.72% |
|  | 0.15 | 0.50 | 0.75 | 0.88 | 1.00 | 0.88 | 0.88 | 0.88 | 0.88 | 0.88 | 0.88 | 0.88 | 1.16% | 1.54% | 1.72% | 1.99% | 1.80% | 1.80% | 1.80% | 1.80% | 1.80% | 1.80% | 1.80% |
|  | 0.16 | 0.38 | 0.63 | 1.00 | 1.00 | 0.88 | 0.88 | 0.88 | 0.88 | 0.88 | 0.88 | 0.88 | 0.78% | 1.35% | 1.91% | 1.91% | 1.72% | 1.72% | 1.72% | 1.72% | 1.72% | 1.72% | 1.72% |
|  | 0.17 | 0.25 | 0.50 | 1.00 | 1.00 | 1.00 | 1.00 | 1.00 | 1.00 | 1.00 | 1.00 | 0.88 | 0.49% | 1.16% | 1.91% | 1.99% | 1.99% | 1.99% | 1.99% | 1.99% | 1.99% | 1.99% | 1.80% |
|  | 0.18 | 0.25 | 0.25 | 0.38 | 0.50 | 0.38 | 0.38 | 0.38 | 0.38 | 0.75 | 0.75 | 0.63 | 0.49% | 0.68% | 0.87% | 1.14% | 0.94% | 0.94% | 0.94% | 0.94% | 1.51% | 1.51% | 1.33% |
|  | 0.19 | **0.00** | 0.25 | 0.25 | 0.50 | 0.38 | 0.38 | 0.38 | 0.63 | 0.63 | 0.50 | 0.50 | **0.00%** | 0.68% | 0.68% | 1.14% | 0.94% | 0.94% | 0.94% | 1.33% | 1.33% | 1.14% | 1.14% |
|  | 0.20 | 0.13 | 0.13 | 0.25 | 0.25 | 0.38 | 0.38 | 0.38 | 0.25 | 0.25 | 0.25 | 0.25 | 0.20% | 0.38% | 0.58% | 0.58% | 0.77% | 0.77% | 0.77% | 0.58% | 0.58% | 0.58% | 0.58% |
|  | 0.21 | 0.25 | **0.00** | 0.25 | 0.25 | 0.38 | 0.38 | 0.38 | 0.25 | 0.25 | 0.25 | 0.25 | 0.40% | **0.00%** | 0.58% | 0.58% | 0.77% | 0.77% | 0.77% | 0.58% | 0.58% | 0.58% | 0.58% |
|  | 0.22 | 0.38 | 0.13 | 0.25 | 0.38 | 0.38 | 0.38 | 0.50 | 0.38 | 0.38 | 0.38 | 0.38 | 0.79% | 0.38% | 0.58% | 0.84% | 0.84% | 0.84% | 1.03% | 0.84% | 0.84% | 0.84% | 0.84% |
|  | 0.23 | 0.50 | 0.00 | 0.25 | 0.25 | 0.13 | 0.25 | 0.25 | 0.25 | 0.25 | 0.25 | 0.25 | 1.00% | 0.00% | 0.58% | 0.58% | 0.38% | 0.58% | 0.58% | 0.58% | 0.58% | 0.58% | 0.58% |
|  | 0.24 | 0.50 | 0.38 | 0.25 | 0.25 | 0.38 | 0.38 | 0.38 | 0.38 | 0.38 | 0.38 | 0.38 | 1.00% | 0.79% | 0.58% | 0.65% | 0.84% | 0.84% | 0.84% | 0.84% | 0.84% | 0.84% | 0.84% |
|  | 0.25 | 0.63 | 0.50 | 0.25 | 0.25 | 0.25 | 0.25 | 0.13 | 0.13 | 0.13 | 0.13 | 0.13 | 1.20% | 1.00% | 0.40% | 0.40% | 0.40% | 0.40% | 0.20% | 0.20% | 0.20% | 0.20% | 0.19% |
|  | 0.26 | 0.75 | 0.50 | 0.13 | 0.13 | 0.25 | 0.25 | 0.38 | 0.38 | 0.25 | 0.25 | 0.25 | 1.41% | 0.81% | 0.20% | 0.26% | 0.46% | 0.46% | 0.66% | 0.66% | 0.46% | 0.46% | 0.46% |
|  | 0.27 | 0.75 | 0.63 | 0.38 | 0.50 | 0.38 | 0.38 | 0.38 | 0.38 | 0.38 | 0.38 | 0.13 | 1.41% | 1.02% | 0.60% | 0.86% | 0.66% | 0.66% | 0.66% | 0.66% | 0.66% | 0.66% | 0.26% |
|  | 0.28 | 0.75 | 0.63 | 0.38 | 0.38 | 0.25 | 0.25 | 0.25 | 0.25 | 0.25 | 0.25 | 0.00 | 1.41% | 1.20% | 0.60% | 0.60% | 0.40% | 0.40% | 0.40% | 0.40% | 0.40% | 0.40% | 0.00% |
|  | 0.29 | 1.00 | 0.75 | 0.63 | 0.75 | 0.75 | 0.75 | 0.63 | 0.75 | 0.63 | 0.63 | 0.63 | 14.75% | 14.14% | 13.93% | 14.20% | 14.20% | 14.20% | 13.99% | 14.20% | 13.99% | 13.99% | 13.99% |
|  | 0.30 | 1.00 | 1.00 | 0.88 | 0.88 | 0.75 | 0.75 | 0.75 | 0.75 | 0.75 | 0.63 | 0.50 | 14.75% | 14.75% | 14.35% | 14.35% | 14.14% | 14.14% | 14.14% | 14.14% | 14.14% | 13.93% | 13.73% |
| *B.subtilis* | 0.10 | **6.75** | 4.13 | 4.25 | 2.88 | 2.38 | 2.13 | 1.88 | 1.13 | **1.00** | 1.25 | 2.00 | **7.85%** | 4.59% | 5.16% | 3.56% | 3.02% | 2.73% | 2.36% | 1.56% | **1.37%** | 1.63% | 2.43% |
|  | 0.11 | 8.00 | 6.75 | 4.75 | 2.88 | 2.38 | 2.13 | 1.88 | 1.75 | 2.00 | 1.38 | 1.13 | 9.61% | 7.89% | 5.37% | 3.17% | 2.58% | 2.27% | 2.02% | 1.85% | 2.09% | 1.44% | 1.17% |
|  | 0.12 | 8.63 | 4.50 | 3.50 | 3.25 | 2.88 | 2.50 | 2.38 | 2.00 | 2.25 | 1.88 | 1.63 | 10.78% | 5.13% | 3.95% | 3.56% | 3.16% | 2.74% | 2.55% | 2.13% | 2.38% | 1.96% | 1.69% |
|  | 0.13 | 9.88 | 6.38 | 4.25 | 3.25 | 2.75 | 2.25 | 2.13 | 2.13 | 2.00 | 1.75 | 1.50 | 12.41% | 7.33% | 4.68% | 3.54% | 2.96% | 2.40% | 2.26% | 2.26% | 2.09% | 1.81% | 1.53% |
|  | 0.14 | 10.00 | 5.75 | 3.63 | 3.88 | 3.75 | 2.75 | 2.00 | 1.63 | 2.00 | 1.75 | 1.50 | 13.13% | 7.06% | 4.59% | 4.86% | 4.70% | 3.62% | 2.77% | 2.31% | 2.69% | 2.43% | 2.17% |
|  | 0.15 | 11.13 | 5.50 | 4.00 | 3.75 | 3.88 | 3.25 | 2.88 | 2.25 | 2.13 | 2.13 | 1.50 | 15.11% | 6.85% | 5.03% | 4.70% | 4.79% | 4.10% | 3.69% | 3.00% | 2.87% | 2.87% | 2.11% |
|  | 0.16 | 11.75 | 6.38 | 6.50 | 4.00 | 4.25 | 3.75 | 3.63 | 3.00 | 2.38 | 2.13 | 2.00 | 16.05% | 7.90% | 8.07% | 4.95% | 5.21% | 4.65% | 4.52% | 3.79% | 3.06% | 2.79% | 2.66% |
|  | 0.17 | 12.13 | 8.25 | 5.38 | 5.00 | 4.75 | 3.88 | 3.63 | 3.13 | 3.13 | 3.13 | 3.00 | 16.56% | 10.22% | 6.61% | 6.13% | 5.83% | 4.79% | 4.51% | 3.96% | 3.97% | 3.97% | 3.83% |
|  | 0.18 | 13.25 | 9.50 | 9.38 | 7.63 | 4.88 | 3.75 | 3.25 | 3.00 | 2.75 | 2.50 | 1.88 | 21.53% | 15.83% | 15.39% | 12.84% | 9.38% | 8.09% | 7.55% | 7.23% | 6.96% | 6.70% | 6.04% |
|  | 0.19 | 13.63 | 7.88 | 7.63 | 5.13 | 5.25 | 4.63 | 3.88 | 3.75 | 3.50 | 3.50 | 3.38 | 22.14% | 13.57% | 12.79% | 9.69% | 9.83% | 9.10% | 8.22% | 8.09% | 7.80% | 7.80% | 7.66% |
|  | 0.20 | 14.50 | 13.00 | 8.63 | 6.25 | 4.38 | 4.50 | 4.13 | 3.50 | 3.38 | 3.13 | 3.13 | 24.16% | 21.41% | 14.77% | 11.76% | 9.56% | 9.70% | 9.32% | 8.59% | 8.43% | 8.14% | 8.14% |
|  | 0.21 | 17.25 | 9.50 | 8.88 | 6.38 | 5.88 | 5.63 | 5.00 | 4.75 | 4.38 | 3.88 | 3.88 | 29.67% | 17.06% | 15.93% | 12.60% | 11.96% | 11.67% | 10.90% | 10.59% | 10.17% | 9.62% | 9.62% |
|  | 0.22 | 18.13 | 10.13 | 7.88 | 6.13 | 5.75 | 5.25 | 4.75 | 5.13 | 5.13 | 4.88 | 4.75 | 32.66% | 19.52% | 16.06% | 13.81% | 13.34% | 12.73% | 12.16% | 12.62% | 12.63% | 12.34% | 12.20% |
|  | 0.23 | 19.25 | 12.13 | 8.75 | 6.75 | 5.75 | 5.88 | 5.88 | 5.25 | 5.00 | 4.88 | 4.88 | 35.06% | 22.31% | 17.09% | 14.65% | 13.40% | 13.50% | 13.49% | 12.73% | 12.44% | 12.31% | 12.31% |
|  | 0.24 | 20.63 | 11.13 | 9.63 | 7.63 | 6.63 | 6.38 | 5.88 | 5.63 | 5.38 | 5.50 | 5.00 | 39.64% | 22.72% | 20.10% | 17.50% | 16.13% | 15.80% | 15.20% | 14.88% | 14.61% | 14.73% | 14.17% |
|  | 0.25 | 22.63 | 17.63 | 14.75 | 9.38 | 7.38 | 6.25 | 6.63 | 6.50 | 6.25 | 6.13 | 5.75 | 46.17% | 35.82% | 31.57% | 22.55% | 19.95% | 18.48% | 18.94% | 18.79% | 18.50% | 18.39% | 17.94% |
|  | 0.26 | 24.75 | 14.50 | 11.25 | 9.63 | 8.25 | 7.88 | 7.63 | 7.38 | 6.88 | 6.50 | 6.50 | 54.93% | 33.52% | 28.65% | 26.44% | 24.58% | 24.02% | 23.65% | 23.36% | 22.73% | 22.28% | 22.32% |
|  | 0.27 | 26.75 | 13.50 | 9.75 | 8.88 | 8.50 | 8.13 | 7.50 | 6.88 | 7.00 | 7.25 | 6.63 | 60.88% | 32.08% | 26.51% | 25.28% | 24.82% | 24.32% | 23.47% | 22.73% | 22.92% | 23.21% | 22.49% |
|  | 0.28 | 28.63 | 22.50 | 20.63 | 13.88 | 9.25 | 8.50 | 7.88 | 7.38 | 7.00 | 6.75 | 6.50 | 69.97% | 53.77% | 50.63% | 37.78% | 31.42% | 30.49% | 29.60% | 28.99% | 28.56% | 28.29% | 27.99% |
|  | 0.29 | 30.50 | 21.75 | 18.25 | 14.25 | 10.50 | 9.88 | 8.63 | 8.25 | 7.88 | 7.75 | 7.50 | 77.80% | 54.97% | 48.45% | 41.97% | 36.91% | 35.97% | 34.33% | 33.82% | 33.39% | 33.24% | 32.99% |
|  | 0.30 | 32.75 | 21.13 | 16.00 | 12.38 | 11.25 | 10.00 | 9.88 | 9.13 | 9.13 | 8.75 | 8.88 | 86.76% | 55.23% | 46.21% | 40.82% | 39.23% | 37.49% | 37.28% | 36.35% | 36.34% | 35.89% | 36.06% |
| *P.aeruginosa* | 0.10 | **1.63** | 1.25 | 1.50 | 1.25 | 1.25 | 0.88 | 1.00 | 1.00 | 1.00 | 1.00 | 1.00 | **5.54%** | 3.75% | 4.17% | 3.39% | 3.28% | 2.39% | 2.74% | 2.74% | 2.74% | 2.74% | 2.74% |
|  | 0.11 | 1.75 | 1.25 | 1.13 | 1.13 | 1.00 | 0.75 | 0.75 | 0.75 | 0.75 | 0.75 | 0.75 | 5.99% | 4.04% | 3.35% | 3.12% | 2.75% | 2.19% | 2.18% | 2.18% | 2.18% | 2.18% | 2.18% |
|  | 0.12 | 1.88 | 1.13 | 1.00 | 1.00 | 0.88 | 0.63 | 0.63 | 0.63 | 0.63 | 0.63 | 0.63 | 6.50% | 3.82% | 3.06% | 3.06% | 2.57% | 1.91% | 1.90% | 1.90% | 1.90% | 1.90% | 1.90% |
|  | 0.13 | 2.13 | 0.88 | 0.88 | 1.00 | 1.00 | 0.75 | 0.63 | 0.50 | 0.50 | 0.50 | 0.50 | 7.49% | 3.02% | 2.77% | 3.06% | 3.06% | 2.28% | 1.91% | 1.54% | 1.54% | 1.54% | 1.54% |
|  | 0.14 | 2.25 | 1.00 | 0.88 | 0.88 | 0.88 | 0.75 | 0.50 | **0.38** | 0.38 | 0.38 | 0.38 | 7.82% | 3.33% | 2.77% | 2.77% | 2.77% | 2.37% | 1.61% | **1.25%** | 1.25% | 1.25% | 1.25% |
|  | 0.15 | 2.50 | 1.38 | 1.00 | 0.88 | 0.88 | 0.75 | 0.50 | 0.50 | 0.50 | 0.50 | 0.50 | 9.20% | 5.13% | 3.85% | 3.46% | 3.37% | 3.07% | 2.34% | 2.34% | 2.34% | 2.34% | 2.34% |
|  | 0.16 | 2.88 | 1.75 | 1.13 | 1.00 | 0.88 | 0.88 | 0.63 | 0.63 | 0.63 | 0.63 | 0.63 | 10.90% | 6.69% | 4.72% | 4.21% | 3.82% | 3.82% | 3.09% | 3.09% | 3.09% | 3.09% | 3.09% |
|  | 0.17 | 3.00 | 1.63 | 1.13 | 1.00 | 0.88 | 0.88 | 0.63 | 0.63 | 0.63 | 0.63 | 0.63 | 11.45% | 6.30% | 4.72% | 4.21% | 3.82% | 3.82% | 3.09% | 3.09% | 3.09% | 3.09% | 3.09% |
|  | 0.18 | 3.25 | 1.75 | 1.25 | 1.13 | 1.00 | 0.88 | 0.63 | 0.63 | 0.63 | 0.63 | 0.63 | 12.52% | 6.73% | 5.12% | 4.60% | 4.21% | 3.82% | 3.09% | 3.09% | 3.09% | 3.09% | 3.09% |
|  | 0.19 | 3.88 | 2.00 | 1.25 | 1.13 | 1.00 | 1.00 | 0.75 | 0.75 | 0.75 | 0.75 | 0.75 | 15.42% | 8.02% | 5.51% | 5.00% | 4.62% | 4.62% | 3.88% | 3.88% | 3.88% | 3.88% | 3.88% |
|  | 0.20 | 4.25 | 2.50 | 1.75 | 1.75 | 1.63 | 1.63 | 1.38 | 1.38 | 1.38 | 1.38 | 1.38 | 17.68% | 10.58% | 8.24% | 8.13% | 7.73% | 7.73% | 6.97% | 6.97% | 6.97% | 6.97% | 6.97% |
|  | 0.21 | 4.75 | 2.38 | 1.63 | 1.50 | 1.25 | 1.25 | 1.00 | 1.00 | 1.00 | 1.00 | 1.00 | 19.85% | 10.07% | 7.65% | 7.13% | 6.35% | 6.35% | 5.61% | 5.61% | 5.61% | 5.61% | 5.61% |
|  | 0.22 | 5.00 | 2.50 | 1.50 | 1.38 | 1.50 | 1.38 | 1.25 | 1.13 | 1.13 | 1.13 | 1.13 | 21.01% | 10.52% | 7.24% | 6.82% | 7.13% | 6.73% | 6.35% | 5.97% | 5.97% | 5.97% | 5.97% |
|  | 0.23 | 5.50 | 3.13 | 2.25 | 2.00 | 1.88 | 1.75 | 1.63 | 1.63 | 1.63 | 1.63 | 1.63 | 23.78% | 13.43% | 10.44% | 9.60% | 9.21% | 8.82% | 8.45% | 8.45% | 8.45% | 8.45% | 8.45% |
|  | 0.24 | 6.00 | 3.25 | 2.38 | 2.00 | 1.88 | 1.75 | 1.63 | 1.63 | 1.63 | 1.63 | 1.63 | 26.39% | 14.44% | 11.45% | 10.30% | 9.91% | 9.52% | 9.15% | 9.15% | 9.15% | 9.15% | 9.15% |
|  | 0.25 | 6.63 | 3.25 | 2.38 | 2.13 | 2.00 | 1.88 | 1.75 | 1.50 | 1.50 | 1.50 | 1.50 | 29.93% | 14.99% | 12.03% | 11.17% | 10.75% | 10.34% | 9.94% | 9.19% | 9.19% | 9.19% | 9.19% |
|  | 0.26 | 7.50 | 3.38 | 2.50 | 2.00 | 1.88 | 1.75 | 1.63 | 1.63 | 1.63 | 1.63 | 1.63 | 33.99% | 15.45% | 12.46% | 10.75% | 10.34% | 9.94% | 9.56% | 9.56% | 9.56% | 9.56% | 9.56% |
|  | 0.27 | 8.13 | 4.75 | 3.75 | 3.13 | 3.00 | 2.88 | 2.75 | 2.75 | 2.75 | 2.75 | 2.75 | 37.43% | 22.29% | 18.67% | 16.64% | 16.23% | 15.83% | 15.45% | 15.45% | 15.45% | 15.45% | 15.45% |
|  | 0.28 | 8.75 | 4.75 | 2.88 | 2.50 | 2.38 | 2.25 | 2.13 | 2.13 | 2.13 | 2.13 | 2.13 | 42.60% | 23.37% | 16.51% | 15.22% | 14.81% | 14.41% | 14.03% | 14.03% | 14.03% | 14.03% | 14.03% |
|  | 0.29 | 9.25 | 5.75 | 3.88 | 3.00 | 2.88 | 2.88 | 2.63 | 2.63 | 2.63 | 2.63 | 2.63 | 45.48% | 25.07% | 18.07% | 15.08% | 14.68% | 14.68% | 13.90% | 13.90% | 13.90% | 13.90% | 13.90% |
|  | 0.30 | 10.00 | 6.38 | 4.13 | 3.50 | 3.38 | 3.13 | 3.00 | 3.00 | 3.00 | 3.00 | 3.00 | 50.57% | 28.34% | 19.79% | 17.65% | 17.22% | 16.39% | 15.99% | 15.99% | 15.99% | 15.99% | 15.99% |
| *E.coli* | 0.10 | **0.00** | **0.00** | 0.00 | 0.00 | 0.00 | 0.00 | 0.00 | 0.00 | 0.00 | 0.00 | 0.00 | **0.00%** | **0.00%** | 0.00% | 0.00% | 0.00% | 0.00% | 0.00% | 0.00% | 0.00% | 0.00% | 0.00% |
|  | 0.11 | 0.00 | 0.00 | 0.00 | 0.00 | 0.00 | 0.00 | 0.00 | 0.00 | 0.00 | 0.00 | 0.00 | 0.00% | 0.00% | 0.00% | 0.00% | 0.00% | 0.00% | 0.00% | 0.00% | 0.00% | 0.00% | 0.00% |
|  | 0.12 | 0.00 | 0.00 | 0.00 | 0.00 | 0.00 | 0.00 | 0.00 | 0.00 | 0.00 | 0.00 | 0.00 | 0.00% | 0.00% | 0.00% | 0.00% | 0.00% | 0.00% | 0.00% | 0.00% | 0.00% | 0.00% | 0.00% |
|  | 0.13 | 0.00 | 0.00 | 0.00 | 0.00 | 0.00 | 0.00 | 0.00 | 0.00 | 0.00 | 0.00 | 0.00 | 0.00% | 0.00% | 0.00% | 0.00% | 0.00% | 0.00% | 0.00% | 0.00% | 0.00% | 0.00% | 0.00% |
|  | 0.14 | 0.00 | 0.00 | 0.00 | 0.00 | 0.00 | 0.00 | 0.00 | 0.00 | 0.00 | 0.00 | 0.00 | 0.00% | 0.00% | 0.00% | 0.00% | 0.00% | 0.00% | 0.00% | 0.00% | 0.00% | 0.00% | 0.00% |
|  | 0.15 | 0.00 | 0.00 | 0.00 | 0.00 | 0.00 | 0.00 | 0.00 | 0.00 | 0.00 | 0.00 | 0.00 | 0.00% | 0.00% | 0.00% | 0.00% | 0.00% | 0.00% | 0.00% | 0.00% | 0.00% | 0.00% | 0.00% |
|  | 0.16 | 0.00 | 0.00 | 0.00 | 0.00 | 0.00 | 0.00 | 0.00 | 0.00 | 0.00 | 0.00 | 0.00 | 0.00% | 0.00% | 0.00% | 0.00% | 0.00% | 0.00% | 0.00% | 0.00% | 0.00% | 0.00% | 0.00% |
|  | 0.17 | 0.00 | 0.00 | 0.00 | 0.00 | 0.00 | 0.00 | 0.00 | 0.00 | 0.00 | 0.00 | 0.00 | 0.00% | 0.00% | 0.00% | 0.00% | 0.00% | 0.00% | 0.00% | 0.00% | 0.00% | 0.00% | 0.00% |
|  | 0.18 | 0.00 | 0.00 | 0.00 | 0.00 | 0.00 | 0.00 | 0.00 | 0.00 | 0.00 | 0.00 | 0.00 | 0.00% | 0.00% | 0.00% | 0.00% | 0.00% | 0.00% | 0.00% | 0.00% | 0.00% | 0.00% | 0.00% |
|  | 0.19 | 0.00 | 0.00 | 0.00 | 0.00 | 0.00 | 0.00 | 0.00 | 0.00 | 0.00 | 0.00 | 0.00 | 0.00% | 0.00% | 0.00% | 0.00% | 0.00% | 0.00% | 0.00% | 0.00% | 0.00% | 0.00% | 0.00% |
|  | 0.20 | 0.00 | 0.00 | 0.00 | 0.00 | 0.00 | 0.00 | 0.00 | 0.00 | 0.00 | 0.00 | 0.00 | 0.00% | 0.00% | 0.00% | 0.00% | 0.00% | 0.00% | 0.00% | 0.00% | 0.00% | 0.00% | 0.00% |
|  | 0.21 | 0.00 | 0.00 | 0.00 | 0.00 | 0.00 | 0.00 | 0.00 | 0.00 | 0.00 | 0.00 | 0.00 | 0.00% | 0.00% | 0.00% | 0.00% | 0.00% | 0.00% | 0.00% | 0.00% | 0.00% | 0.00% | 0.00% |
|  | 0.22 | 0.00 | 0.00 | 0.00 | 0.00 | 0.00 | 0.00 | 0.00 | 0.00 | 0.00 | 0.00 | 0.00 | 0.00% | 0.00% | 0.00% | 0.00% | 0.00% | 0.00% | 0.00% | 0.00% | 0.00% | 0.00% | 0.00% |
|  | 0.23 | 0.00 | 0.00 | 0.00 | 0.00 | 0.00 | 0.00 | 0.00 | 0.00 | 0.00 | 0.00 | 0.00 | 0.00% | 0.00% | 0.00% | 0.00% | 0.00% | 0.00% | 0.00% | 0.00% | 0.00% | 0.00% | 0.00% |
|  | 0.24 | 0.13 | 0.13 | 0.13 | 0.13 | 0.13 | 0.13 | 0.13 | 0.13 | 0.13 | 0.13 | 0.13 | 8.33% | 8.33% | 8.33% | 8.33% | 8.33% | 8.33% | 8.33% | 8.33% | 8.33% | 8.33% | 8.33% |
|  | 0.25 | 0.25 | 0.25 | 0.25 | 0.25 | 0.25 | 0.25 | 0.25 | 0.25 | 0.25 | 0.25 | 0.25 | 11.11% | 11.11% | 11.11% | 11.11% | 11.11% | 11.11% | 11.11% | 11.11% | 11.11% | 11.11% | 11.11% |
|  | 0.26 | 0.25 | 0.25 | 0.25 | 0.25 | 0.25 | 0.25 | 0.25 | 0.25 | 0.25 | 0.25 | 0.25 | 11.11% | 11.11% | 11.11% | 11.11% | 11.11% | 11.11% | 11.11% | 11.11% | 11.11% | 11.11% | 11.11% |
|  | 0.27 | 0.75 | 0.75 | 0.75 | 0.75 | 0.75 | 0.75 | 0.75 | 0.75 | 0.75 | 0.75 | 0.75 | 47.92% | 47.92% | 47.92% | 47.92% | 47.92% | 47.92% | 47.92% | 47.92% | 47.92% | 47.92% | 47.92% |
|  | 0.28 | 0.75 | 0.75 | 0.75 | 0.75 | 0.75 | 0.75 | 0.75 | 0.75 | 0.75 | 0.75 | 0.75 | 47.92% | 47.92% | 47.92% | 47.92% | 47.92% | 47.92% | 47.92% | 47.92% | 47.92% | 47.92% | 47.92% |
|  | 0.29 | 0.88 | 0.88 | 0.88 | 0.88 | 0.88 | 0.88 | 0.88 | 0.88 | 0.88 | 0.88 | 0.88 | 52.38% | 52.38% | 52.38% | 52.38% | 52.38% | 52.38% | 52.38% | 52.38% | 52.38% | 52.38% | 52.38% |
|  | 0.30 | 1.00 | 1.00 | 1.00 | 1.00 | 1.00 | 1.00 | 1.00 | 1.00 | 1.00 | 1.00 | 1.00 | 69.05% | 69.05% | 69.05% | 69.05% | 69.05% | 69.05% | 69.05% | 69.05% | 69.05% | 69.05% | 69.05% |
| *C.albicans* | 0.10 | **1.50** | 0.88 | 0.38 | **0.13** | 0.63 | 0.63 | 0.63 | 0.63 | 0.63 | 0.63 | 0.63 | **12.38%** | 6.18% | 1.90% | **0.34%** | 2.97% | 2.97% | 2.97% | 2.97% | 2.97% | 2.97% | 2.97% |
|  | 0.11 | 1.63 | 1.00 | 0.63 | 0.13 | 0.63 | 0.63 | 0.63 | 0.63 | 0.63 | 0.63 | 0.63 | 13.59% | 7.62% | 3.95% | 0.34% | 2.97% | 2.97% | 2.97% | 2.97% | 2.97% | 2.97% | 2.97% |
|  | 0.12 | 1.63 | 1.00 | 0.63 | 0.38 | 0.50 | 0.50 | 0.50 | 0.50 | 0.50 | 0.50 | 0.50 | 13.59% | 7.15% | 4.40% | 2.29% | 2.37% | 2.37% | 2.37% | 2.37% | 2.37% | 2.37% | 2.37% |
|  | 0.13 | 1.63 | 1.00 | 0.50 | 0.38 | 0.38 | 0.50 | 0.50 | 0.50 | 0.50 | 0.50 | 0.50 | 13.59% | 7.62% | 2.76% | 1.90% | 1.73% | 2.37% | 2.37% | 2.37% | 2.37% | 2.37% | 2.37% |
|  | 0.14 | 1.63 | 1.13 | 0.38 | 0.25 | 0.38 | 0.38 | 0.38 | 0.38 | 0.38 | 0.38 | 0.38 | 13.59% | 8.60% | 1.90% | 1.10% | 1.73% | 1.73% | 1.73% | 1.73% | 1.73% | 1.73% | 1.73% |
|  | 0.15 | 1.63 | 1.00 | 0.50 | 0.25 | 0.38 | 0.38 | 0.38 | 0.38 | 0.38 | 0.38 | 0.38 | 13.59% | 7.15% | 3.10% | 1.10% | 1.73% | 1.73% | 1.73% | 1.73% | 1.73% | 1.73% | 1.73% |
|  | 0.16 | 1.63 | 1.13 | 0.50 | 0.38 | 0.38 | 0.38 | 0.50 | 0.50 | 0.50 | 0.50 | 0.50 | 13.59% | 8.60% | 3.10% | 2.29% | 2.25% | 2.25% | 2.92% | 2.92% | 2.92% | 2.92% | 2.92% |
|  | 0.17 | 1.63 | 0.75 | 0.63 | 0.25 | 0.25 | 0.25 | 0.25 | 0.38 | 0.38 | 0.38 | 0.38 | 13.59% | 5.26% | 4.40% | 1.10% | 1.06% | 1.06% | 1.06% | 1.73% | 1.73% | 1.73% | 1.73% |
|  | 0.18 | 1.75 | 1.13 | 0.63 | 0.38 | 0.25 | 0.25 | 0.25 | 0.25 | 0.25 | 0.25 | 0.25 | 15.39% | 8.60% | 4.40% | 2.29% | 1.06% | 1.06% | 1.06% | 1.06% | 1.06% | 1.06% | 1.06% |
|  | 0.19 | 1.88 | 1.00 | 0.75 | 0.38 | 0.13 | 0.13 | 0.25 | 0.25 | 0.25 | 0.25 | 0.25 | 17.41% | 7.62% | 5.26% | 1.90% | 0.34% | 0.34% | 1.06% | 1.06% | 1.06% | 1.06% | 1.06% |
|  | 0.20 | 2.00 | 1.25 | 0.50 | 0.38 | 0.38 | 0.38 | 0.25 | 0.25 | 0.25 | 0.25 | 0.25 | 19.70% | 9.65% | 3.10% | 2.29% | 2.25% | 2.25% | 1.53% | 1.53% | 1.53% | 1.53% | 1.53% |
|  | 0.21 | 2.38 | 1.00 | 0.75 | 0.50 | 0.25 | 0.50 | 0.38 | 0.38 | 0.38 | 0.38 | 0.38 | 25.04% | 7.62% | 5.26% | 3.10% | 1.53% | 3.10% | 2.29% | 2.29% | 2.29% | 2.29% | 2.29% |
|  | 0.22 | 2.38 | 1.13 | 0.75 | 0.50 | 0.25 | 0.50 | 0.50 | 0.38 | 0.38 | 0.38 | 0.38 | 25.04% | 8.60% | 5.26% | 3.10% | 1.53% | 3.10% | 3.10% | 2.29% | 2.29% | 2.29% | 2.29% |
|  | 0.23 | 2.38 | 1.00 | 0.50 | 0.25 | 0.13 | 0.13 | 0.13 | 0.13 | 0.13 | 0.13 | 0.13 | 26.24% | 8.26% | 4.06% | 1.95% | 0.76% | 0.76% | 0.76% | 0.76% | 0.76% | 0.76% | 0.76% |
|  | 0.24 | 2.50 | 1.00 | 0.63 | 0.25 | 0.38 | 0.38 | 0.38 | 0.38 | 0.38 | 0.38 | 0.38 | 27.92% | 8.26% | 4.92% | 1.95% | 2.75% | 2.75% | 2.75% | 2.75% | 2.75% | 2.75% | 2.75% |
|  | 0.25 | 2.50 | 1.00 | 0.75 | 0.38 | 0.38 | 0.38 | 0.38 | 0.38 | 0.38 | 0.38 | 0.38 | 27.92% | 8.26% | 5.83% | 2.75% | 2.42% | 2.42% | 2.42% | 2.42% | 2.42% | 2.42% | 2.42% |
|  | 0.26 | 2.63 | 1.13 | 0.75 | 0.50 | 0.13 | 0.13 | 0.13 | 0.13 | 0.13 | 0.13 | 0.13 | 30.94% | 9.30% | 5.83% | 3.61% | 0.76% | 0.76% | 0.76% | 0.76% | 0.76% | 0.76% | 0.76% |
|  | 0.27 | 2.63 | 1.25 | 1.00 | 0.63 | 0.25 | 0.38 | 0.38 | 0.38 | 0.38 | 0.38 | 0.38 | 30.94% | 10.43% | 7.86% | 4.52% | 1.56% | 2.42% | 2.42% | 2.42% | 2.42% | 2.42% | 2.42% |
|  | 0.28 | 2.63 | 1.13 | 0.88 | 0.50 | 0.38 | 0.38 | 0.38 | 0.38 | 0.38 | 0.38 | 0.38 | 30.94% | 9.30% | 6.81% | 3.61% | 2.75% | 2.75% | 2.75% | 2.75% | 2.75% | 2.75% | 2.75% |
|  | 0.29 | 2.88 | 1.13 | 0.88 | 0.63 | 0.38 | 0.38 | 0.50 | 0.50 | 0.50 | 0.50 | 0.50 | 34.81% | 9.30% | 6.81% | 4.52% | 2.75% | 2.75% | 3.61% | 3.61% | 3.61% | 3.61% | 3.61% |
|  | 0.30 | 3.00 | 1.38 | 1.00 | 0.75 | 0.50 | 0.50 | 0.50 | 0.75 | 0.75 | 0.75 | 0.75 | 35.73% | 11.35% | 7.74% | 5.45% | 3.68% | 3.68% | 3.68% | 5.45% | 5.45% | 5.45% | 5.45% |

*Table S5. The bacterial species included in the external dataset*

| **Bacteria species** | **ID** | **Gram** | **Culturing** | **Agar** | **Required NAD** | **Required CO₂** | **Selected for counting test** |
| --- | --- | --- | --- | --- | --- | --- | --- |
| *Actinobacillus equuli* | sp01 | − | aerobic | blood |  |  | √ |
| *Actinobacillus pleuropneumoniae* | sp02 | − | aerobic | chocolate | + |  | √ |
| *Aeromonas hydrophila* | sp03 | − | aerobic | blood |  |  | √ |
| *Bacillus cereus* | sp04 | + | aerobic | blood |  |  |  |
| *Bibersteinia trehalosi* | sp05 | − | aerobic | blood |  |  | √ |
| *Bordetella bronchiseptica* | sp06 | − | aerobic | blood |  |  |  |
| *Brucella ovis* | sp07 | − | aerobic | blood |  | + |  |
| *Clostridium perfringens* | sp08 | + | anaerobic | blood |  |  |  |
| *Corynebacterium pseudotuberculosis* | sp09 | + | aerobic | blood |  |  |  |
| *Erysipelothrix rhusiopathiae* | sp10 | + | aerobic | blood |  |  |  |
| *Escherichia coli* | sp11 | − | aerobic | nutrient |  |  | √ |
| *Glaesserella parasuis* | sp12 | − | aerobic | chocolate | + | + | √ |
| *Klebsiella pneumoniae* | sp13 | − | aerobic | blood, nutrient |  |  |  |
| *Listeria monocytogenes* | sp14 | + | aerobic | blood |  |  | √ |
| *Paenibacillus larvae* | sp15 | + | aerobic | blood |  | + |  |
| *Pasteurella multocida* | sp16 | − | aerobic | blood |  |  |  |
| *Proteus mirabilis* | sp17 | − | aerobic | MacConkey |  |  | √ |
| *Pseudomonas aeruginosa* | sp18 | − | aerobic | blood |  |  |  |
| *Rhodococcus equi* | sp19 | + | aerobic | blood |  |  | √ |
| *Salmonella enterica* | sp20 | − | aerobic | nutrient |  |  | √ |
| *Staphylococcus aureus* | sp21 | + | aerobic | blood |  |  | √ |
| *Staphylococcus hyicus* | sp22 | + | aerobic | blood |  |  | √ |
| *Streptococcus agalactiae* | sp23 | + | aerobic | blood |  |  |  |
| *Trueperella pyogenes* | sp24 | + | aerobic | blood |  |  | √ |

*Table S6. Key parameters used for ICC training and evaluation*

| **Parameter** | **Setting** |
| --- | --- |
| Input size | 2048 × 2048 |
| Interaction threshold | τ = 0.2 |
| Configured loss weights | λ_box_ = 0.2, λ_obj_ = 0.5 |
| Optimizer | Adam |
| learning rate | 1×10^-4^; weight decay = 1×10^-4^ |
| Batch size | 8 |
| Training epochs | 60 |
| Checkpoint selection | Highest validation mAP among 60 training epochs |

*Fig. S1. Workflow of the ICC-based annotation tool.*

*Fig. S2. Detailed workflow of ICC model evaluation.*

*Fig. S3. User interface of the ICC-based annotation tool. Supports image upload, target selection, and correction of mistaken detections. The tables display the top-left and bottom-right coordinates of user-provided bounding boxes. Orange and red circles indicate positive and negative user inputs in the current iteration, respectively.*

**Section S2: *B. lactis*-Colony dataset**

1. Bacterial Culture Preparation

The *B. lactis*-Colony dataset uses *Bifidobacterium lactis*. Freeze-dried powder at a concentration of 10^10^ CFU/g was used, with 0.25 g of powder dissolved in 5 mL of MRS liquid medium for revival. After incubation at 37°C for 24 hours, the culture was collected and diluted into different concentration gradients (Table S7). For each concentration gradient, plating was performed with 10 replicate samples per gradient. In each case, 1000 µL of solution was evenly spread onto MRS agar plates using a sterile spreader until the liquid was fully absorbed. This procedure ensured uniform colony distribution on the plates and the acquisition of samples with varying colony densities.

2. Imaging Setup

Colony images were captured using an iPhone 15 Pro. To achieve stable and uniform illumination, a simple lightproof box system was constructed (Fig. S4), with a SupFire GF06 light source. The camera position was fixed during imaging, and images were acquired at resolutions of 3024×3024 and 2700×2700 pixels. There are two lighting conditions applied: (1) diffuse illumination provided solely by a reflective surface, resulting in darker images; (2) standard illumination, approximating typical observational conditions. The original images were in HEIC format and were subsequently converted to JPG format for dataset processing.

3. AI Annotation

Dataset annotation followed the ICC pipeline described in the main text, combining automated predictions with manual corrections. These manual corrections were not independently audited, and the target and non-target categories were assigned primarily based on colony morphology.

*Table S7. Preparation of B. lactis concentration gradients*

| **Concentration relative to stock** | **Volume taken from previous dilution (µL)** | **Volume of MRS medium added (µL)** | **Total volume (µL)** |
| --- | --- | --- | --- |
| 1 | - | - | 5000 |
| 10^-1^ | 100 | 900 | 1000 |
| 10^-2^ | 100 | 900 | 1000 |
| 10^-3^ | 100 | 900 | 1000 |
| 10^-4^ | 100 | 900 | 1000 |
| 10^-5^ | 200 | 1800 | 2000 |
| 3^-1^×10^-5^ | 600 | 1200 | 1800 |
| 3^-2^×10^-5^ | 600 | 1200 | 1800 |
| 3^-3^×10^-5^ | 600 | 1200 | 1800 |
| 3^-4^×10^-5^ | 600 | 1200 | 1800 |

*Fig. S4. Setup of the custom lightproof box for colony imaging*
